# FD and FDP bZIP transcription factors and FT florigen regulate floral development and control homeotic gene expression in *Arabidopsis* floral meristems

**DOI:** 10.1242/dev.204241

**Published:** 2025-05-22

**Authors:** Maida Romera-Branchat, Chloé Pocard, Coral Vincent, Martina Cerise, Vítor da Silveira Falavigna, Alice Pajoro, Na Ding, He Gao, Rainer Franzen, George Coupland

**Affiliations:** Department of Plant Developmental Biology, Max Planck Institute for Plant Breeding Research, Cologne 50829, Germany

**Keywords:** *Arabidopsis*, Flower development, Floral meristem growth, bZIPs, Florigens, FD, FDP, FT, TSF, MADS-domain transcription factors

## Abstract

*Arabidopsis* florigen activation complex (FAC), formed by the interaction of the transcription factor FD and the florigen protein FT, activates gene expression in the shoot apical meristem to induce flowering. We show that FD and its paralog FDP are also expressed in partially overlapping patterns in the floral meristem and floral organs, and that FT is present in floral meristems. The flowers of mutants for FT and its paralog TSF (*ft tsf*), and of *fd fdp* mutants show variable numbers of sepals and petals, and larger floral meristems than wild type. In the floral meristem, *fd fdp* and *ft tsf* mutants show a significant reduction in the expression of SEP and AG genes, which encode MADS-domain transcription factors, as well as increased expression of the homeobox gene *WUS*. Binding of FD to SEP genes suggests that diminished SEP gene expression is a primary defect in the mutants. We conclude that, beyond their role in floral transition, FAC components regulate floral homeotic gene expression to control floral meristem size, and influence floral organ development and identity.

## Teaser

**Summary:** Later in inflorescence development, the florigen transcriptional complex that initiates flowering has important roles in controlling floral meristem and floral organ development.

## INTRODUCTION

Plant shoots are formed from meristems that appear at different stages of plant development. The shoot apical meristem (SAM) is formed during embryogenesis and contains a population of undifferentiated stem cells. These cells are located at the tip of the shoot, and their descendants differentiate on the periphery of the meristem to give rise to organ primordia (Barton, 2010; Fuchs and Lohmann, 2020). During vegetative development of *Arabidopsis*, the primordia on the flanks of the meristem give rise to leaves, and axillary meristems in the axils of these leaves form lateral shoots. Later during shoot growth, and dependent on the environmental conditions, the identity of the SAM changes to an inflorescence meristem, growth of leaf primordia is suppressed, and the identity of axillary meristems change to that of floral primordia that contain floral meristems. The apical inflorescence meristem and floral meristems share aspects of their genetic programs, so that some regulatory genes that confer inflorescence identity on the SAM are also expressed in flowers and contribute to floral development (Gregis et al., 2009; Liu et al., 2009; Xi and Yu, 2009).

In the SAM, FD is expressed during vegetative development and persists throughout the transition to flowering (Abe et al., 2005; Romera-Branchat et al., 2020; Wigge et al., 2005)*.* Moreover, when the plant is exposed to long photoperiods (LDs), mutations in *FD* delay the transition of the SAM from a vegetative to an inflorescence meristem, causing late flowering (Abe et al., 2005; Wigge et al., 2005). The closely related FD PARALOGUE (FDP) protein is expressed below the SAM in a complementary expression pattern to that of FD, and *fdp-CRP* mutations slightly accelerate flowering (Romera-Branchat et al., 2020). FD and FDP physically interact with the florigen proteins FLOWERING LOCUS T (FT) and TWIN SISTER OF FT (TSF) (Abe et al., 2005, 2019; Wigge et al., 2005). The *FT* and/or *TSF* genes are transcribed in the vasculature of leaves in response to LDs and their proteins are transported through the phloem to the SAM where they interact with FD to promote flowering (Adrian et al., 2010; Chen et al., 2018; Corbesier et al., 2007; Jaeger and Wigge, 2007; Mathieu et al., 2007). The interaction between FD and FT is mediated by 14-3-3 proteins to form the florigen activation complex (FAC) (Taoka et al., 2011), which allows transcriptional activation of primary FD targets such as *SUPPRESSOR OF OVEREXPRESSION OF CONSTANS1* (*SOC1*) and *FRUITFULL* (*FUL*), floral meristem identity genes such as *APETALA1* (*AP1*; *AGL7*) and *LEAFY* (*LFY*), which activate each other (Bowman et al., 1993; Wagner et al., 1999), and floral organ identity genes such as those of the SEPALLATA family (SEP) (Collani et al., 2019; Romera-Branchat et al., 2020; Wigge et al., 2005; Zhu et al., 2020). Notably, FD has an additional role in maintaining indeterminacy of the inflorescence meristem by preventing transition of the SAM into a floral meristem. In this role, FD interacts with the FT-related protein TERMINAL FLOWER 1 (TFL1) to repress the expression in the SAM of genes involved in floral development and thereby maintain SAM indeterminacy (Cerise et al., 2023; Goretti et al., 2020; Hanano and Goto, 2011; Zhu et al., 2020).

*Arabidopsis* floral primordia transition through distinct stages from emergence at stage 1 to the mature flower opening at stage 12 (Smyth et al., 1990). During this process, floral organs form in four concentric whorls: sepals initiate at stage 3, petals and stamens at stage 5, and carpel primordia at stage 6. By the end of stage 2, the floral meristem forms centrally, characterized by regulatory genes like *WUSCHEL* (*WUS*) and *CLAVATA3* (*CLV3*) (Fletcher et al., 1999; Long and Barton, 2000; Mayer et al., 1998). The mature flower contains four sepals, four petals, six stamens and two fused carpels. At stage 6, the floral meristem converts into carpels, involving the repression of *WUS* (Lenhard et al., 2001; Lohmann et al., 2001). The identity of each whorl is controlled by homeotic genes within the ABC model, where combinations of A, B and C functions specify sepal, petal, stamen and carpel identities, mainly through MADS-domain transcription factors: AP1 (A function), PISTILLATA (PI) and APETALA3 (AP3) (B function), and AGAMOUS (AG) (C function), which is also involved in termination of growth of the floral meristem by transcriptional repression of *WUS* (Lenhard et al., 2001; Lohmann et al., 2001). Additionally, SEPALLATA MADS-box genes (*SEP1-SEP4*) provide an E function, which is essential for floral organ identity, as inactivation of all four SEP genes converts floral organs into leaves (Ditta et al., 2004).

Here, we have studied the roles of FD, FDP, FT and TSF in *Arabidopsis* floral development. *FT* is expressed in the vasculature of the pedicel and silique of mature flowers, and has been described to maintain inflorescence and floral identity, so that inflorescences of *ft* mutants contain inflorescence nodes that form vegetative structures rather than flowers (Liu et al., 2014; Muller-Xing et al., 2014). We found that FD, FDP, FT and TSF contribute to the regulation of floral meristem size and floral organ development by directly activating SEP gene transcription. Moreover, FD, FDP and FT are present in the floral meristem from floral stage 2-3, where they increase expression of the *SEP2*, *SEP3* and *AG* genes, which is associated with reduced *WUS* expression and floral meristem size regulation. We discuss the significance of the different functions of FT, TSF, FD and FDP at distinct stages of reproductive development to control the identity of the SAM and the growth of floral meristems.

## RESULTS

### *FD* and *FDP* are expressed in partially overlapping domains in the floral meristem and developing floral organs

**Fig. 1.**
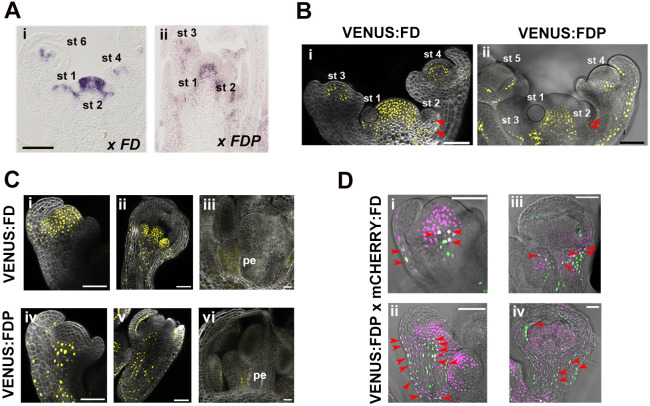
***FD* and *FDP* are expressed in floral organs.** (A) *In situ* hybridization of (i) *FD* and (ii) *FDP* mRNAs in inflorescences of plants grown for 20 long days (LDs) (i) and in 2-week-old plants grown under short days and then transferred to LDs for 9 days (ii). (B) Confocal images of (i) VENUS:FD and (ii) VENUS:FDP in longitudinal views of inflorescences in 18-day-old plants. (C) Confocal images of VENUS:FD floral buds at the end of stage 3 (i), stage 6 (ii) and stage 9 (iii), and VENUS:FDP at stage 3 (iv), stage 5 (v) and stage 9 (vi). pe, petals. (D) Colocalization of VENUS:FDP and mCHERRY:FD in floral buds at stage 3 (i), stage 4 (ii), stage 6 (iii) and stage 7 (iv) of floral development, as visualized by confocal microscopy. Violet signal corresponds to mCHERRY:FD and green signal corresponds to VENUS:FDP. Whiter signal is merged expression of both fluorescent proteins. Red arrowheads indicate colocalization of expression. Scale bars: 100 µm in A-C (i,ii,iv,v); 20 µm in C (iii,vi); 50 µm in D.

*FD* and *FDP* are expressed in floral primordia (Abe et al., 2005; Romera-Branchat et al., 2020; Wigge et al., 2005), but their patterns of expression have not been described in detail. To analyze their spatio-temporal expression patterns in flowers, *in situ* hybridizations were performed on plants grown under short days (SDs) and transferred to long days (LDs) for 9 days (Fig. 1Ai). *FD* mRNA was present at the base of stage 1 and stage 2 floral primordia, and appeared in the floral meristem at stage 3 (Fig. 1Ai, Fig. S1Ai, ii). *FDP* mRNA was detected at stage 3 in the lower floral meristem and adaxial sepals (Fig. 1Aii, Fig. S1Aiii), soon after the formation of the floral meristem at stage 2-3 (Long and Barton, 2000). At stage 3, the spatial patterns of expression of *FD* and *FDP* in the floral meristem are similar to their distribution in the inflorescence meristem (Romera-Branchat et al., 2020).

Higher spatial resolution of *FD* and *FDP* expression patterns was obtained by imaging flowers of *FD::VENUS:FD* and *FDP::VENUS:FDP* transgenic lines (Romera-Branchat et al., 2020) by confocal microscopy. VENUS:FD was present at the base and in the abaxial region of stage 1 and stage 2 floral primordia (Fig. 1Bi). At stage 3-4, VENUS:FD was detected broadly at the floral meristem apex and at the base of sepals (Fig. 1Bi,Ci). At stage 6, VENUS:FD was broadly detected in the first, third and fourth whorls and in the pedicel (Fig. 1Cii), and was expressed throughout developing petal primordia at stage 9 of floral development (Fig. 1Ciii). VENUS:FDP was detected only from stage 2 and 3 at the base of the floral primordia (Fig. 1Bii). From stage 4, it was more broadly detected in the basal part of the floral meristem, in the pedicel, epidermis and adaxial side of the sepals (Fig. 1Civ, v). This pattern was also maintained at stage 6 (Fig. 1Cv). In older flowers (stage 9), VENUS:FDP was also detected at the base of the petals (Fig. 1Cvi). Co-localization of these bZIP transcription factors (TFs) was tested using plants containing both FDP::VENUS:FDP and FD::mCHERRY:FD transgenes (Martignago et al., 2023). VENUS:FDP and mCHERRY:FD were co-expressed in a few cells at the center of the floral meristem at stage 3 and in the epidermis, and this overlap was maintained in older floral buds, including the pedicel cells (Fig. 1Di-iv; Fig. S1Bi-vi).

### *fd fdp* double mutants show defects in floral organ number and identity

**Fig. 2.**
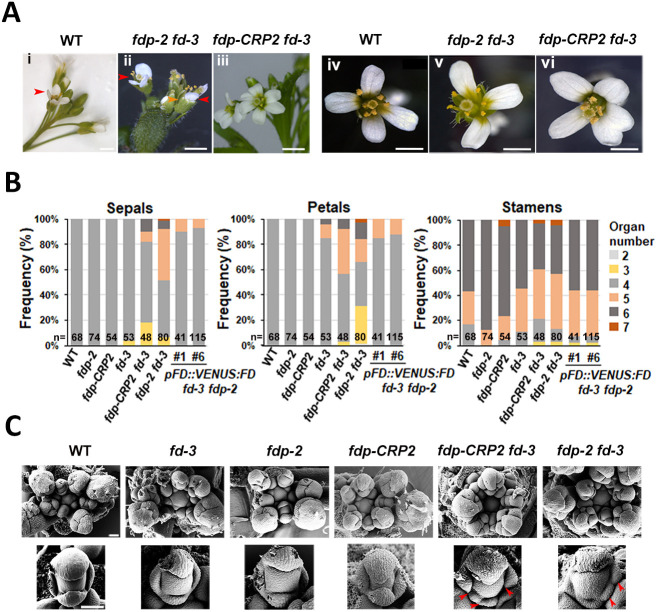
**The *fd fdp* double mutant shows floral organ defects under long-day conditions.** (A) Representative flowers of the genotypes illustrated. The red arrowheads indicate petals and the orange arrowhead indicates sepals. (B) The numbers of sepals, petals and stamens in flowers of the illustrated genotypes. *n* corresponds to the total number of flowers analyzed. (C) SEM images of inflorescences (top) and floral buds (bottom) at stage 4 to 5 of floral development of the genotypes illustrated. Col-0, *fdp-2* and *fdp-CRP2* inflorescences were imaged at day 20, *fd-3* inflorescences at day 30, and *fdp-2 fd-3* and *fdp-CRP2 fd-3* inflorescences at day 34. Red arrowheads indicate sepals with abnormal shapes or growth. Scale bars: 5 mm in A (i-iii); 1 mm in A (iv-vi); 100 µm in C (top); 20 µm in C (bottom).

The floral phenotypes of *fd-3*, *fdp-2* and *fdp-CRP2* single mutants, and double mutants *fd-3 fdp-CRP2* and *fd-3 fdp-2* (Romera-Branchat et al., 2020) were analyzed. The *fdp-2* and *fdp-CRP2* mutations are a dominant missense mutation in the DNA-binding domain and a null frameshift mutation, respectively, and the respective mutant flowers resembled wild-type (Col-0) flowers, with no significant changes in floral organ number (Fig. 2B; Table 1, Table S1). Moreover, *fd-3* mutant flowers showed minor differences in organ number (4% with three sepals, 15% with more than four petals) (Fig. 2B; Table 1, Table S1). However, the double mutants showed a synergistic effect on floral organ number: over 36% and 50% of *fd-3 fdp-CRP2* flowers had altered numbers of sepals or petals, respectively, and over 50% and 65% of *fd-3 fdp-2* flowers had these alterations (Fig. 2A,B, Table S1). Introduction of *FD::VENUS:FD* into *fd-3 fdp-2* largely complemented these defects to Col-0 levels (Fig. 2B, Table 1, Table S1), supporting a partially redundant role for these bZIP TFs in floral development.

**Table 1.** Floral organ number under long day conditions

Genotype	Sepal	Petal	Stamen	Carpel	Total organ number	Number of flowers analyzed
Wild type	4.0±0.0	4.0±0.0	5.9±0.3	2	15.9	151^‡^
*fd-3*	3.9±0.0*	4.1±0.3*	5.6±0.5*	2	15.6	53
*ft-10*	4.4±0.6*	3.8±0.6*	5.4±0.7*	2	15.6	30
*tsf-1*	4.0±0.0^ns^	4.0±0.0^ns^	5.9±0.3^ns^	2	15.9	74
*fdp-2*	4.0±0.0^ns^	4.0±0.0^ns^	5.9±0.4^ns^	2	15.9	74
*fdp-CRP2*	4.0±0.0^ns^	4.0±0.0^ns^	5.7±0.6^ns^	2	15.8	21
*fdp-2 fd-3*	4.5±0.8*	3.7±0.7*	5.5±0.7*	2	15.7	77
*fdp-CRP2 fd-3*	4.1±0.8^ns^	4.5±0.7*	5.3±0.9*	2	15.9	39
*ft-10 tsf-1*	4.0±1.2^ns^	1.7±1.2*	6.1±0.7*	2	13.8	157
*pFDV fdp-CRP2 fd-3 (1)*	4.1±0.4^ns^	4.1±0.5^ns^	5.7±0.8*	2	15.9	41
*pFDV fdp-CRP2 fd-3 (6)*	4.1±0.3^ns^	4.2±0.7*	5.6±0.8*	2	15.9	63

Floral organ number under long day conditions.

ns, not significant (for further details about statistics, see Table S1).

*P<0.05.

^‡^Independent quantifications were applied for the wild type (Col-0).

Young floral buds of *fd-3 fdp-2* and *fd-3 fdp-CRP2* double mutants and of single mutants at stages 4 and 5 were analyzed by scanning electron microscopy (SEM). Notably, only the double mutants exhibited defects in organ development, and formed more than four sepal primordia, unlike Col-0, which always had four (Fig. 2C). In these mutants, adaxial and lateral sepals were smaller, abnormally shaped and more numerous, whereas abaxial sepals appeared similar to Col-0 (Fig. 2C). Older flowers at stages 11, 12 and 13 were also examined (Fig. S2). At stage 13, *fd-3 fdp-CRP2* double mutants displayed a wider pedicel compared with Col-0 (Fig. S2E-G). To quantify this effect, pedicel width at anthesis (stage 13) was then measured for Col-0, *fd-3* and *fdp-CRP2* and for the *fd-3 fdp-CRP2* double mutant*.* Both *fd-3* single and the double mutant showed wider pedicels than the Col-0 (Fig. S2H, Table S2). Additionally, double mutants showed altered organ number and identity, with abnormal sepal shapes and bifurcate trichomes (Fig. S2A-G). Sepaloid cells were also observed on petals (Fig. S2G). These findings suggest that FD and FDP play partially redundant functions in regulating floral organ number and identity.

### *ft-10 tsf-1* double mutants show related but more severe defects in organ patterning and identity than *fd-3 fdp-CRP2* mutants

**Fig. 3.**
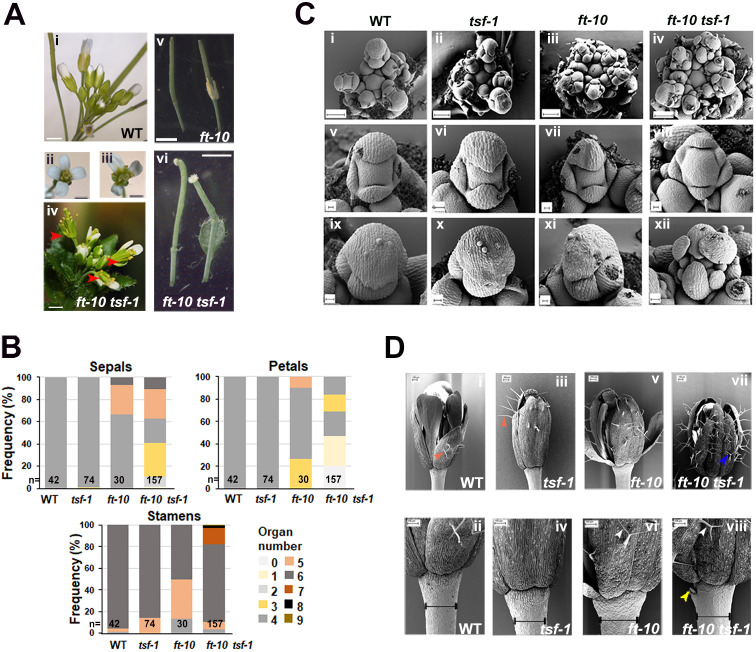
**The *ft tsf* double mutant shows floral defects under long-day conditions.** (A) Floral phenotypes of an inflorescence/flower 30-day-old Col-0 (i,ii) and 45-day-old *ft-10 tsf-1* (iii,iv) plants, showing petal loss (red arrowheads). Carpels in *ft-10* (stage 15 and 16 siliques) resemble Col-0, while *ft-10 tsf-1* carpels have size and number defects (v,vi). (B) Frequency of sepals, petals and stamens in the genotypes shown. *n* indicates the total number of flowers analyzed. (C) SEM images: (i-iv) inflorescences; (v-viii) stage 4 floral buds; (ix-xii) stage 6 floral buds. Col-0 and *tsf-1* at day 25, ft-10 at day 39 and *ft-10 tsf-1* at day 46. (D) SEM images of stage 13 flowers from Col-0 and *tsf-1* (i-iv) and stage 12 from *ft-10* and *ft-10 tsf-1* (v-viii). Orange arrowheads indicate unbranched trichomes (i,iii); white arrowheads indicate branched trichomes (vi and viii in *ft-10* and *ft-10 tsf-1*); blue arrowhead indicates a stellate trichome (vii in *ft-10 tsf-1*); yellow arrowhead indicates stipules on the pedicel (viii in *ft-10 tsf-1*). Pedicel width is indicated by black lines (ii, iv, vi and viii). Scale bars: 2 mm in A (i,iv); 1 mm in A (ii,iii); 1.25 mm in A (v,vi); 100 µm in C (i-iv) and D (ii,iv,vi,viii); 10 µm in C (v,vii,viii,xi); 20 µm in C (vi,ix,x,xii); 200 µm in D (i,iii,v,vii).

The promotion of floral transition by FD depends on FT and TSF in the FAC (Taoka et al., 2011). To investigate the involvement of FT and TSF in floral development, we examined the floral phenotypes of *ft-10*, *tsf-1* and *ft-10 tsf-1* mutants, and compared them to the *fd-3 fdp-CRP2* double mutant (Fig. 3A-D; Fig. S3). The *tsf-1* mutant exhibited a phenotype similar to Col-0, whereas the *ft-10* mutant showed variability in sepal and petal number, with only 70% of flowers developing four sepals and 60% developing four petals (Fig. 3B). However, *ft-10 tsf-1* mutants displayed more severe defects across all four floral whorls, including variable sepal numbers, reduced petal numbers and abnormally shaped carpels (Fig. 3A,B, Table 1, Table S1). While *ft-10 tsf-1* flowers contained an average of 14 floral organs, the *fd-3 fdp-CRP2* double mutant showed a mean of 16 organs, resembling Col-0 plants (Table 1). In the first whorl, *ft-10 tsf-1* mutants exhibited a high degree of variability in sepal number. Only 20% of flowers had four sepals, whereas 40% had fewer than four sepals and the remaining 40% had more than four sepals. In contrast, the *fd-3 fdp-CRP2* double mutant had a higher proportion of flowers with the normal pattern of four sepals, with ∼60% of flowers developing four sepals, 20% having fewer than four, and 20% having more than four sepals (Fig. S3). The second whorl was more severely affected in *ft-10 tsf-1* mutants, with only 15% of flowers developing four petals, while ∼50% had either no petals or just a single petal. In contrast, *fd-3 fdp-CRP2* did not show such a drastic reduction in petal number (Fig. S3). Approximately 50% of *fd-3 fdp-CRP2* flowers had the normal pattern of four petals, as in Col-0, while the rest predominantly developed five petals. This suggests that, while *fd-3 fdp-CRP2* does not fully maintain normal petal numbers, its petal phenotype differs from *ft-10 tsf-1*, where petal loss is a major characteristic.

Further analysis by SEM revealed that *ft-10 tsf-1* floral buds showed more growth defects and disorganized whorls compared with *ft-10* (Fig. 3Ci-iv). At stages 4-5, Col-0 and single mutants had larger abaxial and adaxial sepals than lateral sepals, but in *ft-10 tsf-1*, all sepals were smaller, similar in size and present in higher numbers (Fig. 3Cv-viii). By stage 5-6, sepals fully covered the floral meristem in Col-0 and single mutants, but in *ft-10 tsf-1*, inner whorls remained visible, with sepals that were smaller, variably shaped and present in higher numbers (Fig. 3Cix-xii). Older *ft-10 tsf-1* flowers exhibited leaf-like features, including bifurcate-stellate trichomes on sepals and stipules on the pedicel, both of which were absent in Col-0 and *tsf-1* (compare Fig. 3Di-iv with vii, viii). Measurement of pedicel width showed that *ft-10*, but not *ft-10 tsf-1*, mutants had wider pedicels than Col-0 (Fig. 3ii, iv, vi and viii, Fig. S2H). Conversely, *35S::FT* plants overexpressing *FT* exhibited narrower pedicels (Fig. S2I, Table S2). Bifurcate trichomes were also observed on *ft-10* sepals but less frequently (Fig. 3Dv, vi). Overall, SEM analysis again indicated that the flowers of *ft-10 tsf-1* were more severely affected than those of *fd-3 fdp-2.*

### Floral development of *ft-10 tsf-1* mutants is also impaired under non-inductive short-day conditions

**Fig. 4.**
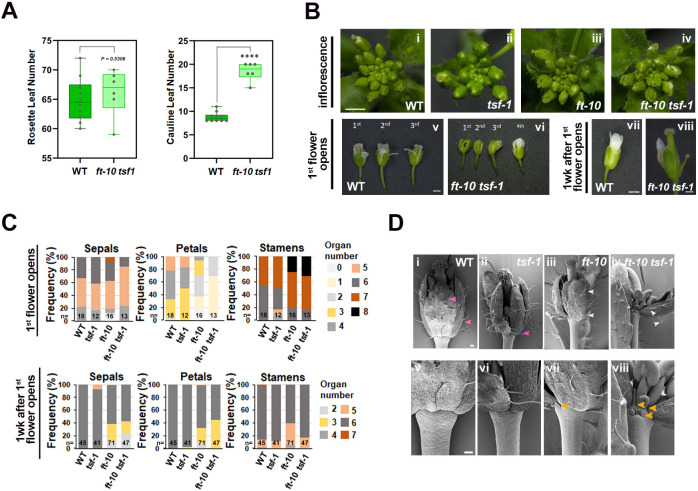
**Floral defects in *ft-10 tsf-1* under short-day conditions.** (A) Flowering time under short-day conditions (SDs) of the Col-0 and *ft-10 tsf-1* plants shown as rosette (left) and cauline leaf numbers (right). Asterisks indicate significant differences (unpaired Student's *t*-test: ****P<0.0001). Box represents the median value and the middle two quartiles of the data, and whiskers represent the remaining two quartiles. (B) Floral phenotypes: top views of inflorescences (i-iv) taken after the first flower had opened in all genotypes. Single flowers (v-viii) for each genotype. (C) Frequency of sepals, petals and stamens for the depicted genotypes. (D) SEM images of stage 13 flowers. Higher magnification views (v-viii) show stipules in *ft-10* (vii) and in *ft-10 tsf-1* (viii) (orange arrowheads). In Col-0 (i,v) and *tsf-1* (ii,vi), most of the trichomes are unbranched (pink arrowheads) (Perazza et al., 1999), but in *ft-10* (iii,vii) and in *ft-10 tsf-1* (iv,viii), trichomes are bifurcated (white arrowheads). Scale bars: 2 mm in B (i-iv); 1 mm in B (v-viii); 200 µm in D (i-iv), 100 µm in D (v-viii).

*FT* transcription in leaves is induced by LDs, but its expression in inflorescences under both LDs and SDs prevents reversion to a vegetative state (Hiraoka et al., 2013; Liu et al., 2014; Muller-Xing et al., 2014). To determine whether *ft-10 tsf-1* plants exhibit floral defects under SDs as well as under LDs, we examined their inflorescence and flower phenotypes under SDs. Flowering time for *ft-10 tsf-1* and Col-0 was similar under SDs when measured by rosette leaf number (Fig. 4A). However, *ft-10 tsf-1* plants produced approximately ten more cauline leaves than Col-0, indicating delayed transition from the I1 phase of inflorescence development to the formation of flowers during the I2 phase (Fig. 4A, Table S3), consistent with previous findings for *ft* mutants (Muller-Xing et al., 2014).

We evaluated flower morphology of the mutants and double mutants under SDs at two stages: when the first flower opened and 1 week later (Fig. 4B). In Col-0 and *tsf-1* mutants, sepals enclosed flower buds, forming a regular spiral pattern in the inflorescence (Fig. 4B, Fig. S4A). In contrast, in *ft-10 tsf-1* double mutants, and to a lesser extent *ft-10* mutants, the flowers were not fully enclosed by sepals, exposing inner whorls and disrupting the regular spiral pattern (Fig. 4Bi-iv, Fig. S4A).

Floral organs in mature flowers were also counted under SDs (Fig. 4C). The flowers of *ft-10 tsf-1* double mutants showed greater variability in organ number compared to Col-0 (Fig. 4Bv-viii). Approximately 60% of *ft-10 tsf-1* mutant flowers had five sepals, and 100% had fewer than four petals, ranging from 0 to 2, whereas 45% of Col-0 flowers had five sepals and 45% had four petals (Fig. 4C, Tables 2, 3, Table S4). One week after the first flower opened on the inflorescence (see Materials and Methods), all Col-0 flowers had four sepals and four petals, but *ft-10* and *ft-10 tsf-1* mutants continued to show altered organ numbers (Fig. 4C, Table 3). Over 40% of *ft-10 tsf-1* flowers had fewer than four sepals and petals (Fig. 4C). Moreover, older flowers of SD-grown *ft-10* and *ft-10 tsf-1* mutants exhibited leaf-like characteristics, such as bifurcated-stellate trichomes on sepals and stipules at the distal part of the pedicel (Fig. 4D). These results demonstrate that FT TSF are also required for floral development under SDs, where they do not control the timing of floral transition.

**Table 2.** Floral organ quantification under short day conditions after the 1st flower opens

Genotype	Sepal	Petal	Stamen	Carpel	Total organ number	Number of flowers analyzed
Wild type	5.1±0.7	3.8±0.8	6.4±0.5	2	17.4	18
*tsf-1*	5.2±0.7^ns^	3.6±0.8^ns^	6.2±0.9^ns^	2	17.2	12
*ft-10*	4.9±1.7^ns^	1.6±1.2*	7.0±0.7*	2	15.7	16
*ft-10 tsf-1*	4.9±0.6^ns^	1.2±0.6*	7.1±0.7*	2	15.3	13

ns, not significant (for further details about statistics, see Table S3).

*P<0.05.

**Table 3.** Floral organ quantification under short day conditions 1 week after 1st flower opens

Genotype	Sepal	Petal	Stamen	Carpel	Total floral organ number	Number of flowers analyzed
Wild type	4.0±0.0	4.0±0.0	5.8±0.4	2	15.8	45
*tsf-1*	4.1±0.3^ns^	4.0±0.1^ns^	5.9±0.3^ns^	2	15.9	41
*ft-10*	3.5±0.7*	3.6±0.7*	5.5±0.6*	2	14.7	71
*ft-10 tsf-1*	3.3±0.6*	3.8±0.6*	5.7±0.7^ns^	2	14.8	47

*P<0.05.

ns, not significant (for further details about statistics, see Table S3).

### The floral meristems of *ft-10 tsf-1* and *fd-3 fdp-CRP2* double mutants are larger than Col-0

**Fig. 5.**
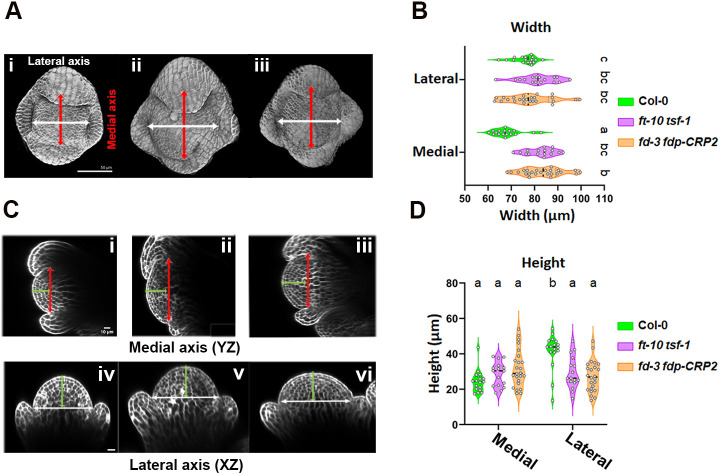
***ft tsf* and *fd fdp* double mutants show defects in floral meristem size and shape.** (A) 3D confocal images of stage 4 floral buds: Col-0 (i), *fd-3 fdp-CRP2* (ii) and *ft-10 tsf-1* (iii). (B) Quantified floral meristem width (*n*: Col-0=17; *ft-10 tsf-1*=16; *fd-3 fdp-CRP2*=24) with significant differences indicated by different letters (one-way ANOVA with Tukey's test; *P*≤0.05). (C) Longitudinal confocal images of stage 4 floral buds of Col-0 (i), *ft-10 tsf-1* (ii) and *fd-3 fdp-CRP2* (iii). White lines depict the width of the lateral axis (iv-vi), red lines depict the width of the medial axis (i-iii) and green lines indicate the height. (D) Quantified floral meristem height. Numbers of flowers measured and statistical analysis was carried out as in B. Scale bars: 50 µm in A; 10 µm in C.

Both FD and FDP are expressed in floral meristems. Therefore, we measured the size and shape of floral meristems using SEM and confocal microscopy. SEM images suggested that floral meristems were larger in *fd-3*, *fd-3 fdp-CRP2* and *ft-10 tsf-1* double mutants compared to Col-0 (Figs 2C and 3C). To quantify these differences, we measured floral meristem dimensions using confocal microscopy. At stage 4, Col-0 floral meristems displayed an elliptical shape, with the lateral axis being wider and taller than the medial axis (Fig. 5A,B, Fig. S4B). However, in the double mutants, the medial axis was significantly wider than in Col-0 (80 µm versus 70 µm), but remained proportional to the lateral axis, effectively eliminating axis differences and resulting in a rounder shape (Fig. 5A,B, Fig. S4B). Additionally, the height of the floral meristems was also more similar between axes in the double mutants, though the lateral axis was reduced compared to Col-0, giving a flatter appearance (Fig. 5C,D, Fig. S4B). These results indicate that FD, FDP, FT and TSF play comparable roles in regulating floral meristem size and shape in stage 3-4 flowers, where *FD* and *FDP* are expressed.

### FT is present in floral meristems where *FD* and *FDP* are expressed

**Fig. 6.**
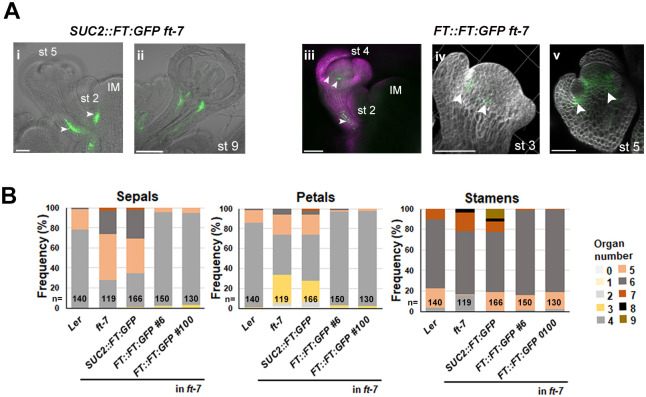
**Presence of FT:GFP in floral meristems and complementation of floral organ defects.** (A) Confocal images of a *SUC2::FT:GFP ft-7* 25-day-old plant (i,ii) and a *pFT::FT:GFP ft-7* 30-day-old plant at various stages (iii-v), showing FT:GFP signal (white arrowheads) in the vasculature (i,ii) and floral meristems (iii-v). (B) Frequency of sepals, petals and stamens for the genotypes shown. *n* indicates the total number of flowers analyzed. Scale bars: 100 µm.

*FT* is transcribed in the mature vasculature of leaves, stems, sepals, pedicels of flowers and siliques (Hiraoka et al., 2013; Liu et al., 2014; Muller-Xing et al., 2014), while FT protein is detected in the vascular tissue of leaves and in the SAM (Abe et al., 2019; Corbesier et al., 2007; Tamaki et al., 2007). To determine if FT is present in developing flowers at stages 3 to 5, as previously shown for FD and FDP, we analyzed *FT::FT:GFP ft-7* and *SUC2::FT:GFP ft-7* reporter lines (Corbesier et al., 2007). The *FT::FT:GFP* transgene contains the endogenous *FT* regulatory sequences, whereas *SUC2::FT:GFP* is specifically expressed in the phloem companion cells. Both transgenes complemented the *ft-7* flowering-time delay (Corbesier et al., 2007) and were expressed in the vascular tissue of developing and mature pedicels (Fig. 6Ai-iii, Fig. S5i-iii). In addition, *FT* transcription was also detected in the pedicel using an *FT::GUS* fusion (Fig. S5iv). FT:GFP expressed from *FT::FT:GFP* was detected in young sepals (stage 4-5) and in the center of the floral meristem at stages 3-5 (Fig. 6Aiii-v). However, when FT was expressed from *SUC2::FT:GFP*, it was not detected in these regions (Fig. 6Ai,ii, Fig. S5i), suggesting that expression in the phloem companion cells does not account for FT protein accumulation in the floral meristem and developing floral organs. Additionally, the *ft-7* mutant in the L*er* background displayed a stronger floral phenotype than *ft-10* in the Col-0 background, with only 30% and 40% of flowers producing four sepals and petals, respectively (Fig. 6B, Table 4, Table S5). This phenotype was fully restored in two independent *FT::FT:GFP ft-7* lines, which showed a floral phenotype similar to wild-type L*er* (Fig. 6B, Table S5), but was not corrected in *SUC2::FT:GFP ft-7* plants. These results suggest that *FT* is expressed in the floral meristem and developing floral organs where the *SUC2* promoter is not active, and that FT protein distribution in flowers overlaps that of FD and FDP.

**Table 4.** Floral organ number under LD conditions

Genotype	Sepal	Petal	Stamen	Carpel	Total organ number	Number of flowers analyzed
Wild type (Ler)	4.2±0.4	4.1±0.4	5.9±0.7	2	16.3	140
*ft-7*	5.2±0.8*	4.1±1.1^ns^	6.1±0.7*	2	17.4	119
*pSUC2::FT:GFP* in *ft-7*	5.0±0.9*	4.0±1.0^ns^	6.2±1.1*	2	17.2	166
*pFT::FT:GFP* in *ft-7 #6*	4.0±0.3^ns^	4.0±0.3^ns^	5.8±0.8^ns^	2	15.9	150
*pFT::FT:GFP* in *ft-7 #100*	4.0±0.3^ns^	4.0±0.2^ns^	5.8±0.5^ns^	2	15.8	130

*P<0.05.

ns, not significant (for further details about statistics, see Table S4).

### *WUS* expression is increased in stage 6 and 8 floral buds of *fd-3 fdp-CRP2* and *ft-10 tsf-1* double mutants compared with those of Col-0

The *fd-3 fdp-CRP2* and *ft-10 tsf-1* mutants have larger floral meristems than Col-0, and FD, FDP and FT proteins are present in floral meristems; therefore, the expression of genes that regulate floral meristem size was examined in these mutants. Floral meristem size is controlled by the CLV/WUS feedback loop, and determinacy of the floral meristem is conferred by repression of *WUS* transcription by AG (Lenhard et al., 2001; Lohmann et al., 2001).

**Fig. 7.**
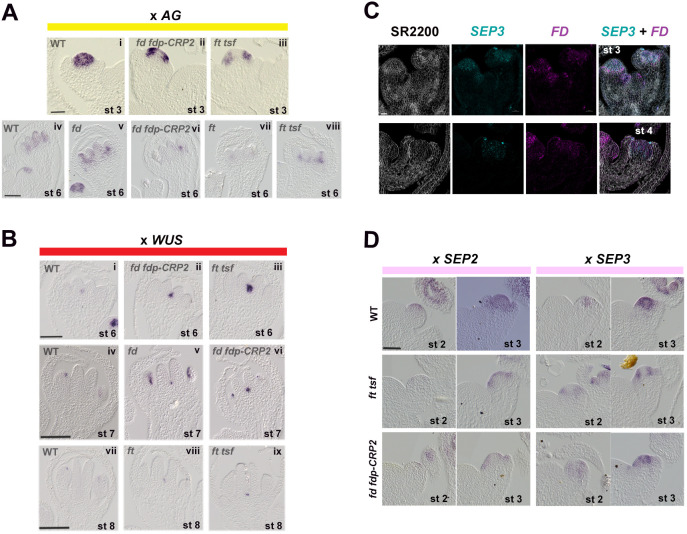
**FT TSF and FD FDP positively activate SEP genes and *AG*, and repress *WUS* in developing flowers.** (A) *In situ* hybridization of *AG* mRNA in inflorescences of the depicted genotypes. Flower buds shown were at the indicated stages. Plants were grown for 24 LDs for Col-0, for 28 LDs for *fd-3*, for 33 LDs for *fd-3 fdp-CRP2*, for 35 LDs for *ft-10* and for 45 LDs for *ft-10 tsf-1.* (B) *In situ* hybridizations of *WUS* mRNA in inflorescences of the depicted genotypes. Flower buds shown were at the indicated stages. Plants were grown for 24 LDs for Col-0, for 28 LDs for *fd-3*, for 31 LDs for *fd-3 fdp-CRP2*, for 35 LDs for *ft-10* and for 40 LDs for *ft-10 tsf-1.* (C) RNAscope *in situ* hybridizations of *SEP3* and *FD* mRNAs in inflorescences of Col-0. SCRI Renaissance 2200 stains cell walls in white. *SEP3* and *FD* mRNAs appear in the depicted colors. Flower buds shown are at stage 3 and stage 4. Plants were grown for 18 LDs. (D) *In situ* hybridizations of *SEP2* and *SEP3* mRNAs in inflorescences of the depicted genotypes. Flower buds shown were at the indicated stages. Plants were grown for 19 or 23 LDs for Col-0, for 27 or 31 LDs for *fd-3 fdp-CRP2*, and for 37 or 45 LDs for *ft-10 tsf-1*. Images show flower buds at stages 2 and 3. Scale bars: 50 µm in A,B,D; 20 µm in C.

To understand the contributions of FD, FDP and FT to the larger floral meristems observed in the mutants, we analyzed *AG* and *WUS* expression by *in situ* hybridization in *fd-3*, *fd-3 fdp-CRP2*, *ft-10* and *ft-10 tsf-1* mutants (Fig. 7A,B, Fig. S6). In Col-0, *AG* was expressed throughout the floral meristem from stage 3-4, but in *fd fdp-CRP2* and *ft-10 tsf-1* mutants, *AG* mRNA was present at the boundaries of the floral meristem but weak or absent in the center (Fig. 7Ai-iii, upper panels, Fig. S6A). By stage 6, *AG* mRNA was detected in the developing carpels and stamens of Col-0 flowers, and was reduced in flowers of *ft-10*, *ft-10 tsf-1* and *fd-3 fdp-CRP2* at the same stage, but remained similar to Col at this stage in the *fd-3* single mutant (Fig. 7iv-viii). Therefore, FD, FDP and FT are required for *AG* expression in floral organs at this stage.

Next, we examined *WUS* mRNA by *in situ* hybridization at stages 6-8. In flowers of Col-0, *WUS* expression was reduced to a small region at stage 6 and was not detected in the center of stage 7 or 8 flowers (Fig. 7Bi,iv,vii). In stage 6 *ft-10 tsf-1* or *fd-3 fdp-CRP2* flowers, *WUS* expression was detected more strongly than in Col-0 (Fig. 7Bi-iii). Moreover, in flowers of *fd-3*, *ft-10*, *fd-3 fdp-CRP2* and *ft-10 tsf-1*, *WUS* persisted for longer into stages 7 and 8 (Fig. 7Bi-ix, lower panels; Fig. S6B). This increased and extended pattern of *WUS* expression is consistent with the reduced *AG* expression and the enlarged floral meristems observed in these mutants.

### FD and FT increase SEP gene expression in floral primordia

SEP MADS-domain transcription factors activate *AG* transcription (Castillejo et al., 2005; Liu et al., 2009), and *sep* mutants are defective in floral organ number (Biewers, 2014) and produce flowers with leaf-like features (Ditta et al., 2004). Given the similarities between these phenotypes and the floral defects observed in *ft-10 tsf-1* and *fd-3 fdp-2* or *fd-3 fdp-CRP2* mutants, we analyzed the expression levels of all four SEP genes (*SEP1*, *SEP2*, *SEP3* and *SEP4*) by RT-qPCR using RNA extracted from inflorescences of *ft-10*, *tsf-1*, *ft-10 tsf-1*, *fd-3*, *fdp-CRP2* and *fd-3 fdp-CRP2* mutants (Fig. S7). In *ft-10*, *tsf-1* and *ft-10 tsf-1* mutants, the mRNA levels of *SEP1*, *SEP2*, *SEP3* and *SEP4* were reduced, with the strongest reductions observed for *SEP2* and *SEP3* mRNAs (fourfold and fourfold lower, respectively, in *ft-10 tsf-1*) (Fig. S7). Similarly, in *fd-3 fdp-CRP2* double mutants, all four *SEP* gene mRNAs were also reduced, though less than in *ft-10 tsf-1* (Fig. S7). To determine whether the defects observed in all four floral whorls could also be attributed to reduce expression of ABC genes within the floral organ identity model, we examined the expression of *AP1*, *PI*, *AP3* and *LFY* (as a C class gene) by qRT-PCR across all mutant combinations. However, no significant differences in their expression were detected in *ft-10 tsf-1* and *fd-3 fdp-CRP2* double mutants (Fig. S7). These data indicate that, among the floral development genes tested, FT TSF and FD FDP predominantly regulate the expression of the four SEP genes, which likely contributes to the observed floral defects in the respective mutants.

The effect of FT TSF and FD FDP on the pattern of expression of *SEP2* and *SEP3* in the floral meristem was then examined. Notably, RNAscope analysis of mRNA colocalization confirmed that the spatial expression pattern of *SEP3* mRNA broadly overlapped with that of *FD* throughout the floral meristem from stages 3 to 5 (Fig. 7C and Fig. S8A). *SEP3* mRNA expression was then compared in Col-0, *ft-10 tsf-1* and *fd-3 fdp-CRP2* by *in situ* hybridization. As previously described (Mandel and Yanofsky, 1998), *SEP3* mRNA was first detected in stage 2 floral buds of Col-0, and this early expression pattern was similar in Col-0, *ft-10 tsf-1* and *fd-3 fdp-CRP2* (Fig. 7B). Later, at stage 3, *SEP3* mRNA was present throughout the floral meristem of Col-0, but in *ft-10 tsf-1* was present only on the flanks of the floral meristem, similar to the pattern described earlier for *AG*, and in *fd-3 fdp-CRP2 SEP3* mRNA was detected in a more restricted pattern than in Col-0 towards the apex of the floral meristem (Figs 1, 3 and 7D, Fig. S8B, upper panels). *In situ* hybridization also showed that *SEP2* mRNA, which appears in Col-0 floral primordia at stage 2 (Savidge et al., 1995), was either absent or more narrowly expressed in *ft-10 tsf-1* and *fd-3 fdp-CRP2* mutants at the same stage (Fig. 7D; Fig. S8B, upper panels). At stage 3, *SEP2* mRNA was detected in the entire floral meristem dome and sepal primordia of Col-0, but was unevenly distributed and restricted to the first three layers in the mutants (Fig. 7D, Fig. S8B, upper panels). We also analyzed the expression pattern of *SEP3* in stage 4 and stage 6 flowers of Col-0 and different mutants by *in situ* hybridization (Fig. S8B). Compared with Col-0, *SEP3* mRNA was reduced in the *ft-10* single mutant to a similar extent as in *ft-10 tsf-1* double mutants*.* By contrast, *SEP3* expression in *fd-3* mutants resembled that of Col-0, suggesting that both FD and FDP activate *SEP3* (Fig. S8B). These experiments demonstrate that FT and FD FDP are required for the spatial expression pattern of *SEP2* and *SEP3* in the Col-0 floral meristem. In agreement with the RT-qPCR results, *LFY* mRNA showed a similar expression pattern by *in situ* hybridization in Col-0, *ft-10 tsf-1* and *fd-3 fdp-CRP2* (Fig. S8B).

### FD binds to SEP genes and shares target genes with ABC MADS-domain transcription factors

**Fig. 8.**
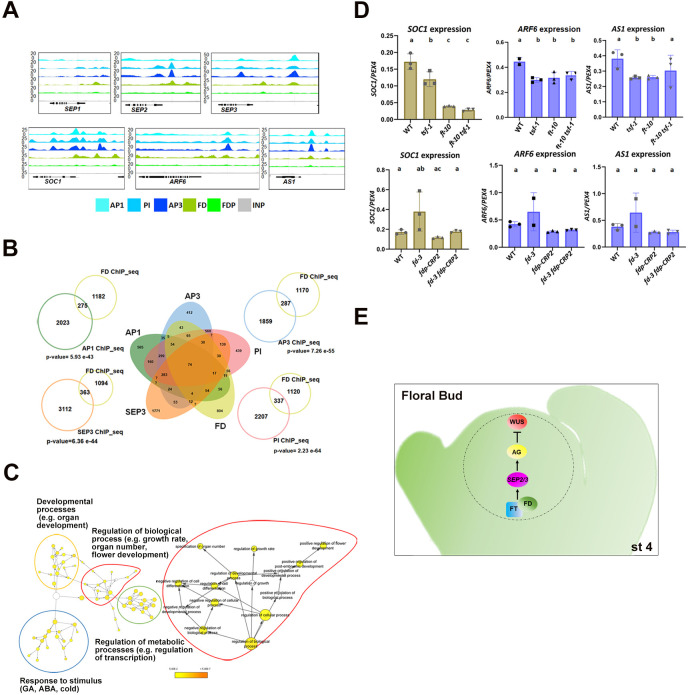
**Comparison of FD ChIPseq gene targets with the targets of other floral organ identity MADS-box transcription factors.** (A) Binding profiles of FD and MADS box transcription factors to *SEP1*, *SEP2* and *SEP3*, and other genes involved in flower development. The panels display, from the top, AP1, PI, AP3, FD, FDP and control (INPUT-gray) peaks at the loci shown, visualized with the IGB browser. (B) Venn diagrams comparing the common target genes in ChIP-seq datasets for FD and AP1, PI, AP3 and SEP3. For the comparison between two datasets (FD versus MADS-box transcription factors), the *P*-values of the overlaps were calculated using a one-sided Fisher's exact test. (C) The GO terms enriched among the 74 genes bound by FD and AP1, AP3, PI and SEP3. The image on the left shows an overview of all GO terms (labels have been removed for a clearer depiction of the different clusters); on the right is a more detailed view for the GO term ‘Regulation of biological process’. (D) qRT-PCR analysis of the mRNA abundance of *SOC1*, *ARF6* and *AS1* in inflorescences of Col-0, *tsf-1*, *ft-10* and *ft-10 tsf-1* double mutants (top), and Col-0, *fd-3*, *fdp-CRP2* and *fd-3 fdp-CRP2* (bottom). Common letters among genotypes indicate no significant differences in mRNA levels (one-way ANOVA followed by Tukey's multiple comparisons test). Different letters indicate statistically significant differences at *P*≤0.05. (E) Model depicting the regulation of floral meristem size and determinacy by the FT and FD FDP proteins, as described in the text. FD FDPs are also expressed in other floral organs, and their effects on floral organs likely also involve the activation of SEP gene transcription, but the precise regulatory mechanisms remain unknown.

The genomic binding sites of FD and FDP were identified by ChIP-seq (Collani et al., 2019; Romera-Branchat et al., 2020; Zhu et al., 2020). Analysis of the ChIP-seq data showed that *SEP2* and *SEP3* were bound by FD, suggesting that their activation by FD, as detected by *in situ* hybridization and RT-qPCR may be a direct effect. Similarly, *SEP1* was also bound by FD (Fig. 8A).

The target genes of several MADS-domain TFs contributing to floral development have also been identified by ChIP-seq (Kaufmann et al., 2009, 2010; Wuest et al., 2012). Comparing FD targets with those of AP1, PI, AP3 and SEP3 revealed 74 genes bound by these four MADS-domain TFs and FD (Fig. 8B, Table S6). GO-term analysis of this subset showed enrichment in processes related to organ development, floral development, growth rate and floral organ number specification (Fig. 8C). Among the identified targets, *SEP2*, *SEP3*, *LFY*, *AP1* and *SOC1*, which all encode TFs that contribute to floral development, were bound by FD and all of the MADS-domain proteins (Table S6). RT-qPCR analyses showed that, among these, only *SEP2* and *SEP3* were reduced in expression in both *ft tsf* and *fd fdp* genotypes (Fig. S7), although *SOC1* was also reduced in *ft tsf* (Fig. 8E). Similarly, *ASYMMETRIC LEAVES 1* (*AS1*) and *AUXIN RESPONSE FACTOR 6* (*ARF6*), which act in parallel to negatively regulate floral organ growth (Tabata et al., 2010), were bound by FD (Fig. 8A) and all of the MADS-domain proteins. *AS1* mRNA was reduced in *ft* mutants and *ARF6* expression was reduced in *ft tsf* (Fig. 8E). These DNA-binding and RT-qPCR data suggest a direct role for FD in regulating organ growth.

## DISCUSSION

This study demonstrates that the FAC components FD, FDP, FT and TSF contribute to floral development independently of their roles in promoting floral transition. These functions begin at stage 3 of floral development, and influence floral meristem shape and size, organ number and identity, and organ growth. FD, FDP, FT and TSF activity in the center of the floral meristem increases transcript levels of regulatory MADS-domain TFs, including all four SEP proteins and AG, and reduces *WUS* expression. This highlights that FT proteins and the FAC complex formed with FD are crucial not only for initiating floral transition and floral primordium identity but also for later stages of floral development.

### Expression of FD, FDP, FT and TSF during flower development

Prior to floral development, *FD* and *FDP* are expressed in the vegetative and inflorescence SAM; we find that in the stage 1 flower they are expressed at the base of the primordium and at stage 1-2 on the abaxial side of the primordium (Romera-Branchat et al., 2020). Later during flower development, FD appears in the meristematic region of stage 2-3 flowers and FDP is expressed from stage 3 onwards in a domain mainly below that of FD, so that their patterns of expression in the floral meristem resemble those in the inflorescence meristem (Romera-Branchat et al., 2020). From stage 3 of floral development, their patterns of expression in the floral meristem partially overlap, but *fd* single mutants already show persistent *WUS* expression, suggesting that FD has a major effect in the floral meristem. Later, as floral organs develop, the domains of FD and FDP expression overlap in sepals, petals and pedicel tissues, consistent with their partially redundant effects on floral organ number. *FT* is transcribed in the mature phloem of several shoot tissues, including leaves, inflorescence stems, pedicels and sepals (Adrian et al., 2010; Kardailsky et al., 1999; Kobayashi et al., 1999; Liu et al., 2014; Muller-Xing et al., 2014). Moreover, to initiate floral transition, FT protein moves from the phloem tissue to the shoot apex, where it interacts with FD to promote flowering gene transcription (Abe et al., 2019; Corbesier et al., 2007; Jaeger and Wigge, 2007; Mathieu et al., 2007). Using an *FT::FT:GFP* transgenic plant (Corbesier et al., 2007), we detected FT protein in the meristematic region of stage 3-4 flowers. This result indicates that FT, FD and FDP are present in the same regions of the floral meristem, and therefore, in addition to its role in the SAM, the FAC is likely formed in the floral meristem to regulate meristem size and determinacy (Fig. 8E). However, this role of FT does not depend on expression of *FT* in the phloem, and therefore most likely not on the movement of FT protein from the vasculature, because a *SUC2::FT:GFP* transgene that strongly drives FT protein expression in the phloem and complements the late-flowering phenotype of *ft* mutants (Corbesier et al., 2007) does not complement the floral phenotype of *ft*. We propose, therefore, that *FT* is transcribed directly in developing flowers to produce FT protein that can interact with FD and FDP, and form the FAC to regulate floral development. *FT* mRNA has not so far been detected in flowers at this stage, but might be present at extremely low levels and/or transiently, and may not necessarily be transcribed in the cells in which FT protein is present, because it may move locally within the flower. FT expression in flowers might be photoperiod-independent, since similar floral defects were observed under both LDs and SDs. Photoperiod-independent expression of *FT* in the inflorescence has been detected previously (Liu et al., 2014; Muller-Xing et al., 2014), although its spatio-temporal pattern of expression in developing floral buds was not determined. Therefore, *FT* expression in flowers may be controlled by a different set of upstream regulators than has been defined in the phloem.

### Effects of FD, FDP, FT and TSF in flower organ development

The *fd* and *fdp* mutations have a synergistic effect on floral organ development that, together with their overlapping expression patterns, argues for a partially redundant effect of the genes at this stage. The double-mutant phenotypes include variable floral organ number, floral homeotic changes and wider pedicels. Some features, such as bifurcated trichomes on sepals and the presence of stipules on the pedicel are characteristic of a reversion to a leaf-like vegetative phenotype (Ditta et al., 2004). The effects of *ft tsf* on floral organ number were stronger than those of *fd fdp*, as previously described for flowering-time control (Martignago et al., 2023; Romera-Branchat et al., 2020). This effect was particularly evident in the strong reduction in petal number in *ft-10 tsf-1* mutants, which was not found in *fd-3 fdp-2* or *fd-3 fdp-CRP2* mutants, despite the FD and FDP proteins being expressed in young petal primordia. This observation suggests that other Group A bZIP TFs might also act during flower development and contribute additional layers of genetic redundancy with FD and FDP, as found in the SAM in the promotion of flowering (Martignago et al., 2023).

The phenotypic effects of *fd fdp* and *ft tsf* in floral development correlate with reduced expression of floral homeotic genes that are direct targets of FD, namely the class E MADS-domain SEP genes. Analysis by RT-qPCR in developing floral buds showed that the reduction in SEP gene transcription was stronger in *ft tsf* than in *fd fdp*, which correlates with the stronger effects on floral organ development discussed above. Moreover, *SEP3* mRNA was reduced to a lesser extent than *SEP2* and this might reveal layers of redundancy with other floral regulators in the regulation of *SEP3*, e.g. transcript levels of *LFY* and *AP1* were not significantly reduced in the inflorescences of *fd fdp* or *ft tsf* and they have been shown to affect SEP gene expression (Kaufmann et al., 2010; Winter et al., 2011). SEP proteins are present in many different tetrameric MADS-domain protein complexes (de Folter et al., 2005; Honma and Goto, 2001), and reduced SEP gene expression not only results in homeotic changes, as predicted by its function in floral organ identity, but also the single or double combination of *sep* mutants affects floral organ number (Biewers, 2014) in a similar way to that observed in *fd fdp* and *ft tsf* mutants. How SEP genes regulate floral organ number is not yet clear, but might involve many MADS domain TF complexes. Thus, our study reveals a new layer of regulation of floral organ number by FT TSF, and to a lesser extent FD FDP, that is required for the formation of protein complexes including SEP proteins. Floral organ number is also more severely affected in *ft tsf* than in *fd fdp* mutants. Sepals of *ft tsf* mutants are smaller and grow asynchronously, exposing the floral meristem and inner whorls, suggesting a role for FT and TSF in sepal size robustness (Burda et al., 2024). Moreover, *ARF6* and *AS1* were downregulated in *ft tsf*, and their mutants display smaller organs in the first three whorls (Tabata et al., 2010), similar to the effect of *ft tsf* on sepals.

### Role of FD, FDP, FT and TSF on floral meristem size and shape

The floral meristems of *fd-3 fdp-CRP2* and *ft-10 tsf-1* double mutants were larger, flatter and more symmetrical than those of Col-0 flowers. The almost identical defects in floral meristem size and shape in *ft tsf* and *fd fdp* supports the idea that both classes of protein act in the FAC to control common processes during floral development. *In situ* hybridization showed that *SEP2* and *SEP3* mRNAs are reduced in the floral meristem in *fd fdp* and *ft tsf* mutants, at stage 3-4 when FD, FDP and FT are present. Floral meristem size is likely regulated independently of organ number, because *fd fdp* and *ft tsf* had altered floral meristems of similar size and shape but different effects on floral organ numbers. Moreover, in these FAC component double mutants, *AG* expression was strongly reduced in the center of the floral meristem, whereas on the flanks of the floral meristem it can be expressed more independently of the FAC. This experiment reveals a spatial pattern of regulation of *AG* transcription during the early stages of carpel development. The role of FD, FDP, FT and TSF in activating *AG* is likely to be indirect, probably through the SEP proteins, because direct binding to several SEP genes was detected but no direct binding of FD and FDP to *AG* was found (Collani et al., 2019; Romera-Branchat et al., 2020; Zhu et al., 2020), and SEP proteins have previously been shown to activate *AG* transcription (Castillejo et al., 2005; Liu et al., 2009). We propose therefore that the primary function of the FAC in the floral meristem is the activation of SEP genes, as shown schematically in Fig. 8E. This model could now be tested by exploring the effects of expressing SEP genes from *FD* regulatory sequences in the *fd-3 fdp-CRP2* and *ft-10 tsf-1* double mutants.

The larger floral meristem of *fd fdp* and *ft tsf* also correlated with higher levels of *WUS* expression persisting into later stages of floral development. This effect on *WUS* is probably an indirect result of reduced *AG* expression in the center of the floral meristem of *ft tsf* and *fd fdp* mutants, because repression of *WUS* transcription by AG has been shown to contribute to floral determinacy (Lenhard et al., 2001; Lohmann et al., 2001). Similarly, larger floral meristem size due to persistent *WUS* expression may contribute to the greater number of organs in inner whorls observed, e.g. in an *ft soc1 agl24 svp* background (Xi and Yu, 2009), although we did not observe more stamens in *ft tsf* double mutants, indicating that the increase in stamen number in *ft soc1 agl24 svp* is a synergistic effect of these mutations rather than being caused only by an increase in *WUS*. Nevertheless, *ft tsf* mutants show a larger floral meristem and a reduction in the total number of floral organs, suggesting that FT and TSF regulate floral organ number by mechanisms other than increasing floral meristem size.

### Perspectives

The precise spatio-temporal expression pattern of *FT* in flowers and whether it is regulated by known floral regulators remain to be determined. This analysis is complicated by the extremely low level of *FT* mRNA and the possibility that it is only transiently expressed in small groups of cells. Moreover, the *FT* regulatory sequences are complex, and the observation that the floral phenotype is present in non-inductive SDs suggests that CONSTANS, which in leaves is activated only under LDs (Suarez-Lopez et al., 2001), may not be involved in the floral expression of *FT*, although this remains to be tested. Similarly, mutations in orthologs of *FT* have been described to cause floral phenotypes in other species (Molinero-Rosales et al., 2004), and, in these cases, whether the temporal and spatial patterns of expression we describe in *Arabidopsis* for FT, FD and FDP are conserved remains to be determined.

In the SAM, FT activity is antagonized by the closely related protein TFL1; in *tfl1* mutants, FT and FD activity is increased at the SAM (Ahn et al., 2006; Cerise et al., 2023; Hanano and Goto, 2011; Zhu et al., 2020). *TFL1* is not expressed in flowers (Bradley et al., 1997; Ratcliffe et al., 1999), suggesting that, in these tissues, FT can interact with FD and activate target genes independently of competition with TFL1. This effect would therefore be comparable to the determinate SAM phenotype of the *tfl1* mutant because, in the mutant, TFL1 is absent at the tip of the SAM and therefore FT can carry out a comparable role to that in Col-0 flowers by activating genes required for floral organ development. Thus, the activity of FT and FD at the SAM in *tfl1* mutants may be comparable to that we have described here for FT in the floral meristem.

## MATERIALS AND METHODS

### Plant growth conditions

In this study, Col-0 and Landsberg *erecta* (L*er*) accessions were used as wild type. Seeds were stratified on soil for 2-4 days in the dark at 4°C and were then transferred to controlled environmental conditions at 20-21°C under long-day conditions (LDs) (16 h light/8 h dark) or short-day conditions (SDs) (8 h light/16 h dark) under a photosynthetic photon flux density of ∼180 μmol s^−1^ m^−2^.

All mutant and transgenic lines used in this study: *fdp-2*, *fd-3*, *fdp-CRP2*, *fd-3 fdp-CRP2*, *fd-3 fdp-2*, *ft-10*, *tsf-1*, *ft-10 tsf-1*, *ft-10 tsf-1*, *fdp-2 fd-3*, *ft-7*, *pFD::VENUS:FD* in *fdp-2 fd-3*, *pFD::VENUS::FD* in *fd-3*, *pFDP::VENUS:FDP* in *fdp-2*, *pFT::GUS*, *pFD:mCHERRY:FD* in *fd-3*, *GAS1:FT*, *GAS1::FT* in *ft-10 tsf-1*, *SEP3::GFP*, *SUC2::FT:GFP* in *ft-7* and *FT::FT:GFP* in *ft-7* have been previously described (Adrian et al., 2010; Andres et al., 2015; Corbesier et al., 2007; Jang et al., 2009; Martignago et al., 2023; Romera-Branchat et al., 2020; Torti et al., 2012; Urbanus et al., 2009).

### Floral organ quantification

To score floral organs under LDs, the floral organs of all open flowers at stage 12-13 (Smyth et al., 1990) on the primary inflorescence were counted on 3-4 consecutive days until between 25 and 150 flowers were scored in total from at least five plants per genotype. Each experiment was performed independently at least twice.

To score floral organs under SDs, measurements were performed at two time points to capture any effects associated with the age of the inflorescence. First, the floral organs were counted for the first flower to open on the primary inflorescence on a total of 19 plants in Col-0, 12 in *tsf-1*, 18 in *ft-10* and 13 in *ft-10 tsf-1*. One week later, a second measurement was performed by counting the floral organs of all flowers at stage 12-13 using a total of 45 flowers for Col-0, 41 in *tsf-1*, 73 for *ft-10* and 47 for *ft-10 tsf-1.*

### Pedicel width analysis

For each genotype, flowers at anthesis (stage 13 according to Smyth et al., 1990) were harvested from the primary shoot from ∼15 plants and observed under a binocular microscope. Pedicel width was manually measured from flower images using ImageJ software (Schindelin et al., 2012). The width of each pedicel was measured twice from the same image and each experiment was independently performed twice.

### Scanning electron microscopy analysis

Inflorescences were dissected manually after plants had bolted and fixed overnight in FAA [50% (v/v) ethanol, 5% (v/v) acetic acid, 1.85% (w/v) formaldehyde]. Samples were dehydrated through an ethanol series and were critical-point dried in liquid CO_2_. Leaf primordia on the dried shoot apex samples were removed under a binocular microscope. The prepared samples were mounted on stubs, sputter-coated with gold and palladium and subjected to high-resolution scanning electron microscopy (Zeiss, Supra 55 VP FEG) with a Gatan Alto 2500 cryo system. More than five shoot apices for each genotype were analyzed.

### Confocal microscopy

Shoot meristems of *pFD::VENUS::FD* in *fd-3* and *pFDP::VENUS:FDP* in *fdp-2* were dissected manually and incubated overnight in 4% (w/v) paraformaldehyde (Sigma-Aldrich) before being treated with ClearSee for 2–4 days as previously described (Kurihara et al., 2015). Samples were stained with SCRI Renaissance 2200 dye (Musielak et al., 2015) for a minimum of 4 h to overnight, and were imaged using a Zeiss LSM780 confocal laser scanning microscope. Minor modifications were performed when FT:GFP protein was observed from *ft-7* mutant lines carrying *SUC2::FT:GFP* or *FT::FT:GFP*; in particular, fixation was performed only for 1 h and samples were incubated in ClearSee solution overnight. The settings used to detect VENUS fluorescent protein have been described previously (Romera-Branchat et al., 2020). To detect mCHERRY, the excitation wavelength was 561 nm and detection wavelength was 570-650 nm.

### GUS staining

GUS staining was performed as described previously (Sessions et al., 1999) except that samples were not treated with acetone. After overnight incubation in GUS-staining solution, samples were dehydrated through an ethanol series and mounted on slides in Hoyer's solution (Hedhly et al., 2018).

### *In situ* hybridization

To analyze the mRNA localization of *FD* and *FDP* genes, plants were grown for 3 weeks in SDs and were then transferred to LDs for 3, 5 or 7 days. To detect *SEP2*, *SEP3*, *WUS*, *AG* and *LFY* mRNAs, Col-0 plants were grown for 19LD or 23LD, *fd-3 fdp-CRP2* mutants were grown for 27LD or 31LD, and *ft-10 tsf*-1 plants were grown for 37LD or 45LD. The samples were inflorescence apices harvested at ZT3. The *in situ* hybridizations were performed as described previously (Torti et al., 2012). The probes used to amplify *SEP2*, *SEP3*, *WUS*, *AG* and *LFY* genes are listed in Table S7. Probes for *FD* and *FDP* were described previously (Romera-Branchat et al., 2020).

### RNAscope fluorescent multiplex assays

The RNAscope assay was conducted following the RNAscope Multiplex Fluorescent Assay v2 protocol provided by ACDBio (materials and method are available at https://acdbio.com/rnascope-multiplex-fluorescent-v2-assay). Briefly, formalin-fixed, paraffin-embedded (FFPE) tissue samples were used for analyses. Probes for FD and SEP3 were custom designed by ACDBio and were assigned to channels C1 and C3, respectively, with the following catalog numbers: 1307011-C1 (FD) 15 pair specific probe, target region 230-1064 of NCBI Reference sequence NM_119756.5; and 1569931-C3 (SEP3)16 pair specific probe, target region 392-1268 of NM_180622.3. RNAscope 3-plex Negative Control Probe (catalog number 320871) was used as a negative control. The protocol was followed exactly except that probes were hybridized overnight at 40°C.

To visualize the targets, TSA Plus fluorophores (diluted in TSA buffer from ACDBio) were applied as follows: TSA Vivid 520 (323271, diluted 1:2500) for C3 and TSA Vivid 570 (323272, diluted 1:1500) for C1. Additionally, Renaissance (0.1% v/v in PBS, 5 min at room temperature) was used to stain the cell walls.

Confocal images were captured using a Zeiss LSM 880 confocal microscope. The Renaissance signal was detected at 410-503 nm with an excitation wavelength of 405 nm. The filter settings for FITC and Cy3 were used for the TSA Vivid Fluorophore 520 and 570 separately.

### RNA extraction and qRT-PCR analysis

Total RNA was extracted with the RNeasy kit (QIAGEN) from the whole primary inflorescence (containing between 2 mm and 3 mm of stem) 2-4 days after the opening of the first flower of plants grown under LDs and was treated with DNA-free DNase (Ambion). For reverse transcription, 2 mg RNA was used following the manufacturer's instructions (Invitrogen). For each replicate, 4-6 inflorescences were harvested. To quantify the expression level of floral genes, cDNAs were synthesized with SuperScript IV (Invitrogen) and qRT-PCR was performed on the Bio-Rad CFX384 using iQ SYBR Green Supermix (Bio-Rad). Each sample was run in technical triplicate and three biological replicates were performed per genotype. For the quantification, the delta-delta CT method was used, the *EIF4* gene was used as a reference gene to normalize expression. Primers used for qRT-PCR are listed in Table S7.

### Quantification of floral meristem size

Shoot apices of Col-0, *fd-3 fdp-CRP2* and *ft-10 tsf-1* plants were harvested at 18LD, 27LD and 34LD, respectively, to sample the first ten flowers initiated by each genotype. Samples were prepared for confocal laser scanning microscopy, as described in the confocal analysis section. All images were analyzed using Fiji software (Schindelin et al., 2012). To obtain images, flowers at stage 4 were identified, reoriented to an upright position, and the height and width of the floral meristem was measured after identifying the medial and lateral axes of sepal formation, according to Smyth et al. (1990). Statistical analyses were performed using two-way ANOVA followed by Tukey's multiple comparison test (GraphPad, www.graphpad.com).

### Target comparisons of ChIP-seq and GO analysis

A list of MADS-domain-protein target genes wase obtained from previously published data: AP1 (Kaufmann et al., 2010), SEP3 (Kaufmann et al., 2009), and PI and AP3 (Wuest et al., 2012). A list of bound regions in the genome was obtained using the CSAR pipeline (van Mourik et al., 2015). To assign the corresponding target gene, a genomic interval from 3 kb upstream the start codon to 1 kb downstream the stop codon was considered. The list of FD targets was the same as previously identified (Romera-Branchat et al., 2020).

For GO analysis, the BiNGO tool (Maere et al., 2005) was used, and the results were visualized using the Cytoscape platform. The color of the circles represents the *P*-value and the size represents the over-representation or under-representation of GO categories according to a hypergeometric test [false discovery rate (FDR)<0.05]. For the comparison of genes bound by FD with those bound by MADS-domain transcription factors (AP1, PI, AP3 and SEP3), Fisher's one-sided exact test was used.

### Quantification and statistical analysis

For graph representation, GraphPad PRISM software was used. When several data points were compared, an ANOVA and Kruskal–Wallis one-way ANOVA on Ranks was used. Different letters indicate statistically significant differences at *P*≤0.05. For simpler two-way comparisons, a non-parametric *t*-test was used. Asterisks indicate differences among two genotypes.

## Supplementary Material



10.1242/develop.204241_sup1 Supplementary information

Table S1. Quantification of floral organ number in long days for genotypes using Col-0 background

Table S2. Pedicel width

Table S3. Flowering time under short days

Table S4. Quantification of floral organ number in short days for genotypes using Col-0 background

Table S5. Quantification of floral organ number in long days for genotypes us ing Ler background

Table S6. 74 genes bound by FD and MADS-box transcription factors.

Table S7. List of primers used in this study
