# Supplementary material for: FD and FDP bZIP transcription factors and FT florigen regulate floral development and control homeotic gene expression in Arabidopsis floral meristems
Source: Development. 2025 May 22;152(10):dev204241. doi: 10.1242/dev.204241 (PMC12148031; doi:10.1242/dev.204241)
Supplement: Supplementary information [file develop-152-204241-s1.pdf]

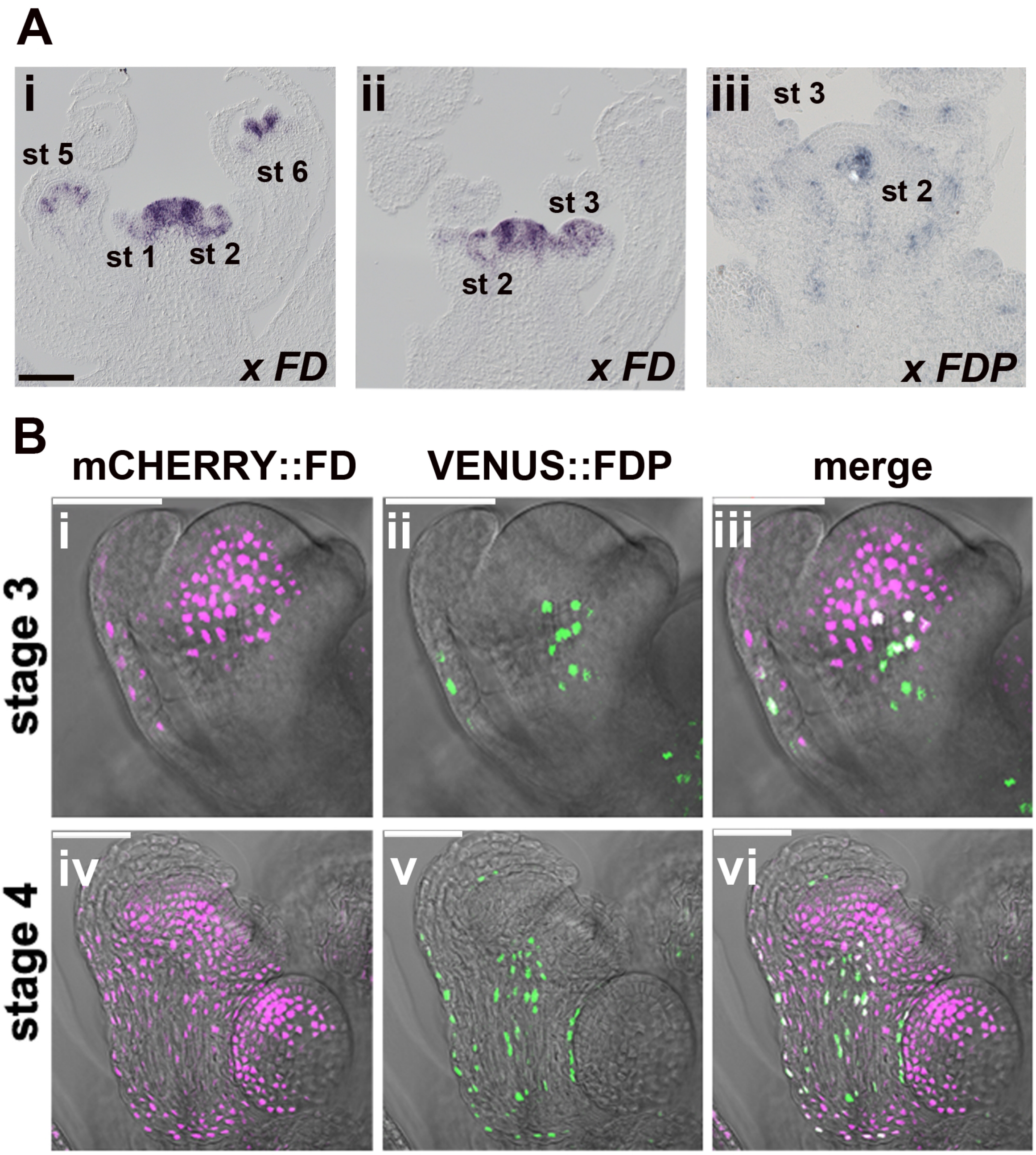

Fig. S1. Related to Fig. 1. Expression patterns of FD and FDP in floral buds.
(A) *In situ* hybridization of (i, ii) *FD* and (iii) *FDP* mRNAs in inflorescences of plants grown for 24 long days (LDs) and 2-week-old plants grown under short days and transferred to LDs for 9 days respectively. (B) mCHERRY:FD and VENUS:FDP colocalize in floral buds at stage 3 and 4 of floral development. White signal is the co- expression of both fluorophores. These are the same images as shown in Figures 1Di and 1Dii, but here the mCherry:FD (i, iv) and VENUS:FDP (ii, v) signals are shown separately as well as together (iii, vi). Scale bars A = 100 µm; B = 50 µm.

Development • Supplementary information

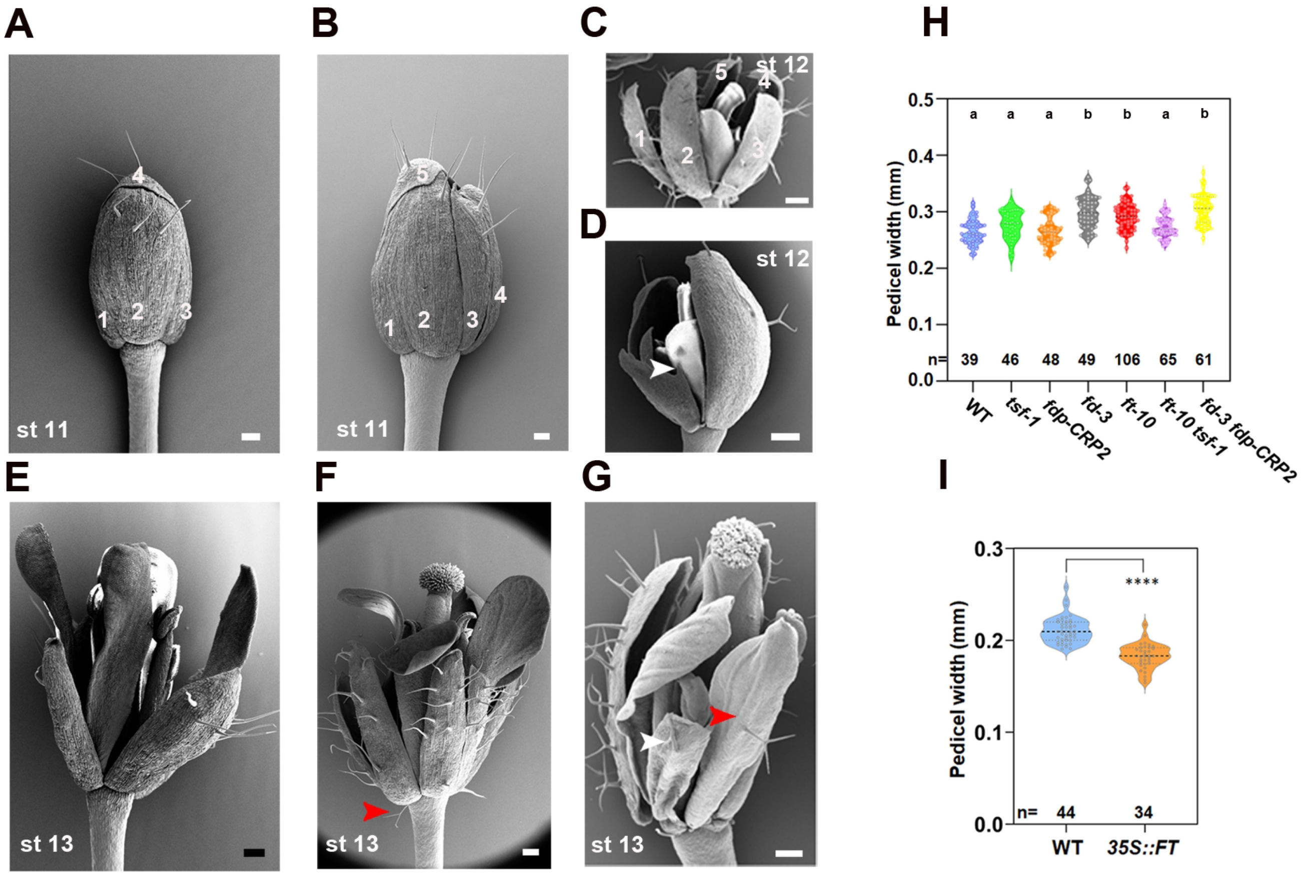

Fig. S2. Related to figure 2. Floral homeotic changes in the double mutants *fd-3 fd-CRP2* and *fd-3 fdp-2* and quantification of the pedicel length. SEM images of wild-type flowers at stage 11 (A) and 13 (E), *fd-3 fdp-CRP2* flowers at stage 11 (B) and 13 (F) and *fd-3 fdp-2* flowers at stage 12 (C, D) and 13 (G). Numbers in A, B and C mark sepals. The white arrowhead in D highlights an abnormally shaped sepal that is smaller, more pointed and with serrated edges. The red arrowhead in F marks a stellate trichome that is typical of leaves, also observed in G. The white arrowhead in G marks an abnormal sepal that shows homeotic transformation into a chimeric sepaloid petal. (H, I) Pedicel width at anthesis (stage 13). In (H), shared letters indicate no significant differences; different letters denote significant differences (one- way ANOVA with Tukey's test; $P \leq 0.05$). In (I), asterisks indicate significant differences between WT and *35S::FT* (Student's t test: ****, $P < 0.0001$). Scale bars in panel A, B and F = 100 μm; in C, D, E and G = 200 μm.

Development • Supplementary information

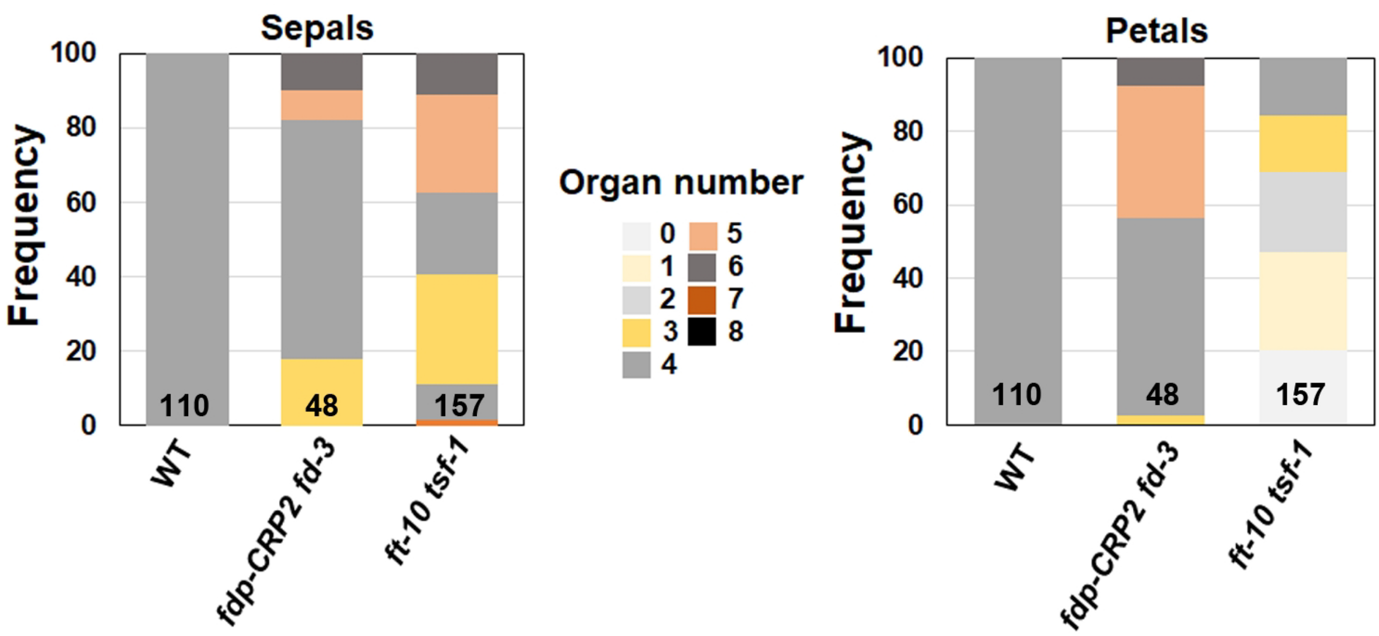

Fig. S3. Related to Fig. 2 and Fig. 3. Diagram comparing number of sepals and petals in *fd fdp* and *ft tsf* double mutants. Numbers of sepals and petals in flowers of the illustrated genotypes. The value “n” corresponds to the total number of flowers analysed.

Development • Supplementary information

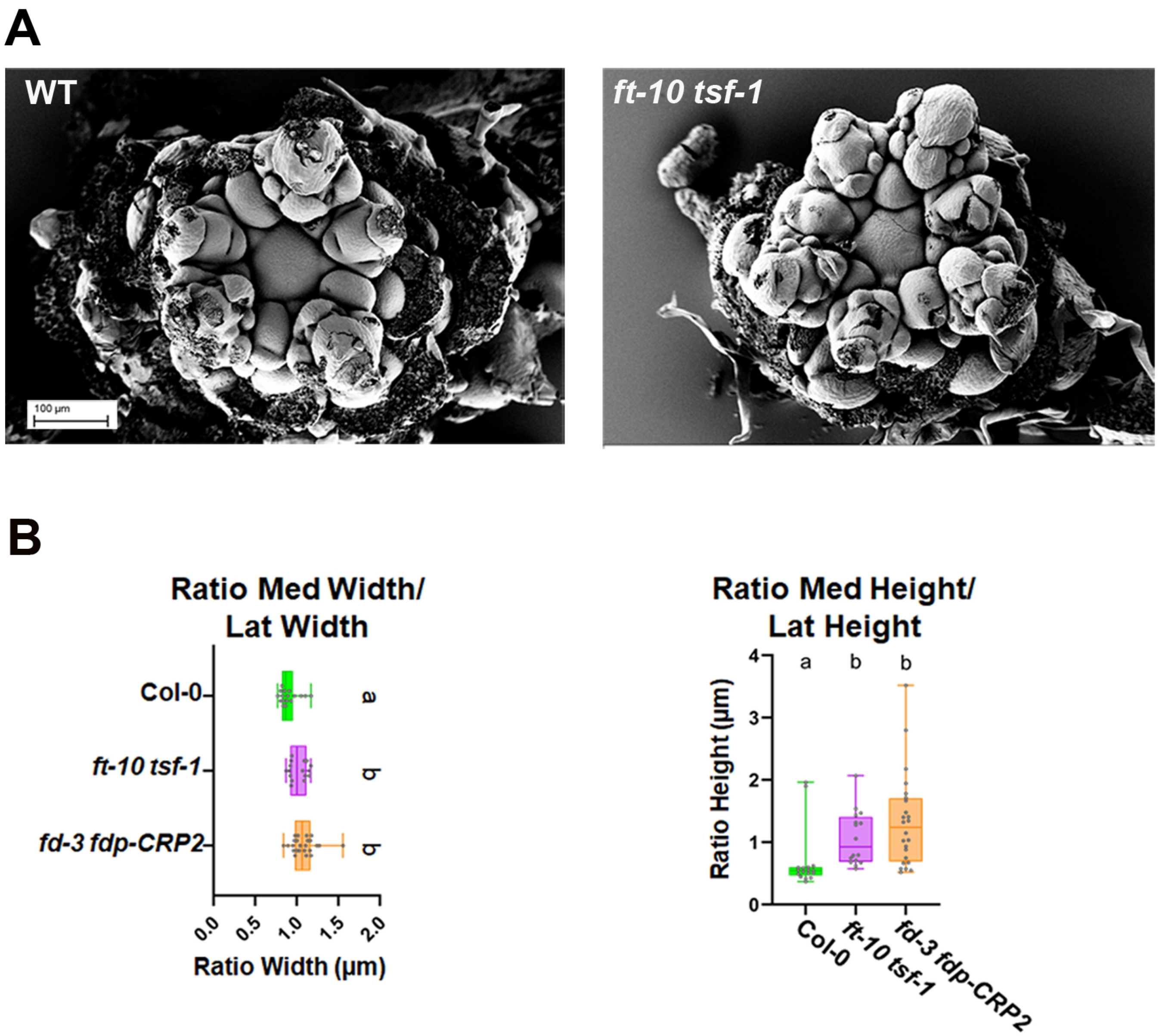

Fig. S4. Related to Fig. 4 and Fig. 5. SEM of inflorescences of WT and *ft tsf* growing under SDs and floral meristem growth analysis of WT, *fd fdp* and *ft tsf* double mutants. (A) SEM images of inflorescences of 7-week-old WT plants, and 9 week-old *ft-10 tsf-1* plants grown under short days. Plants were harvested after the first flower had opened for each genotype. Note that almost all floral buds in *ft tsf* showed defects in organ patterning and number. (B) The ratio of medial sepal/lateral sepal width and the medial sepal/lateral sepal height for stage 4 floral buds of the genotypes depicted. Number of floral meristems (n) for WT = 17; *ft-10 tsf-1*, n = 16; *fd-3 fdp-CRP2*, n = 24. Common letters among genotypes indicate no significant differences in floral meristem width or height. Different letters indicate statistically significant differences in a one-way ANOVA followed by Tukey's multiple comparison test ($P \leq 0.05$). Scale bar A= 100 µm.

Development • Supplementary information

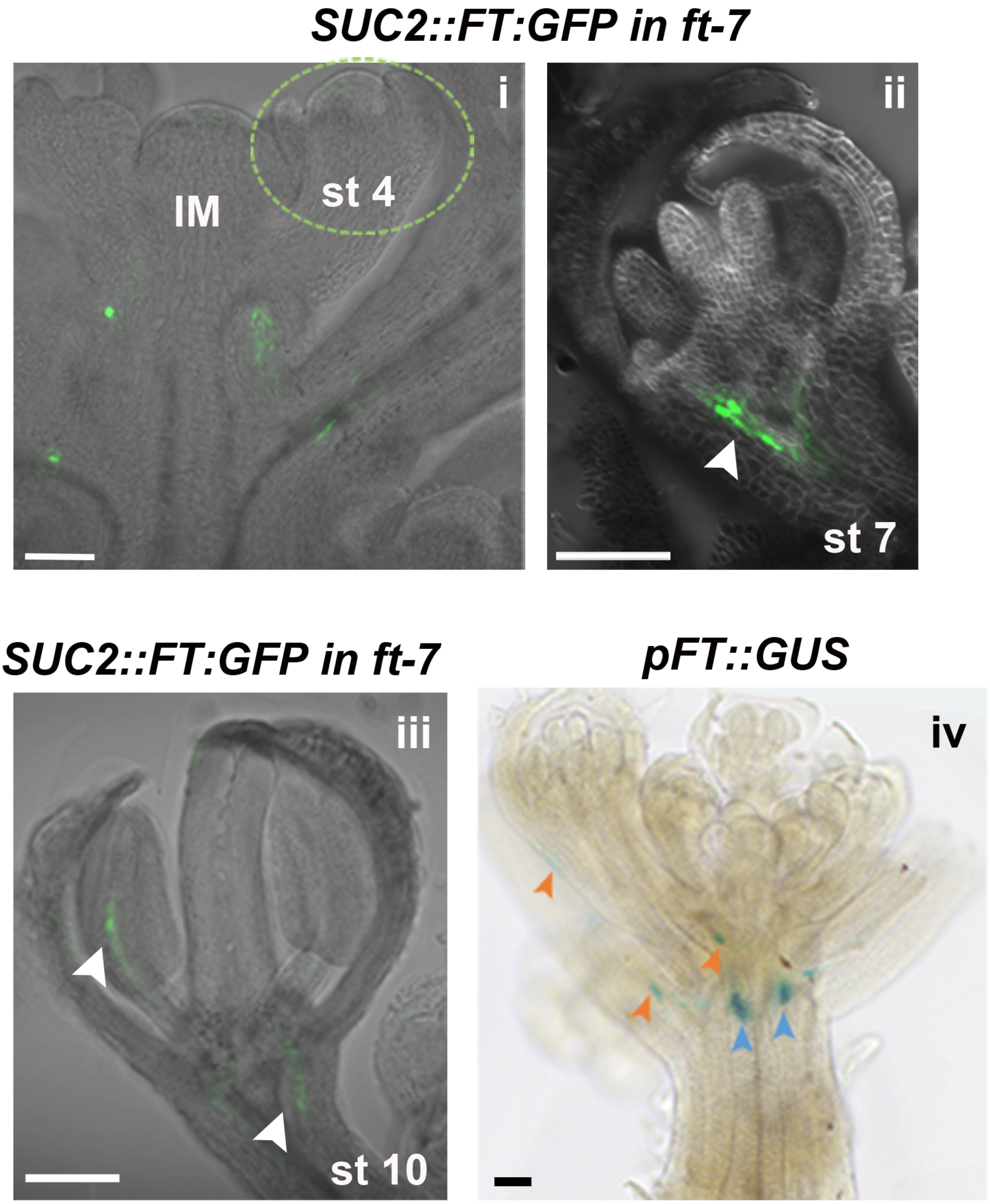

Fig. S5 related to Fig. 6. *FT* mRNA and FT protein expression from the *SUC2* promoter in inflorescences and in flowers. (i, ii, iii). Confocal images of FT:GFP fluorescence in *SUC2::FT:GFP ft-7* inflorescence shoot apex (i) and floral buds (ii), (iii). IM represents the inflorescence meristem. In i, the green dashed circle highlights the absence of FT protein in the floral meristem. White arrowheads point to FT:GFP signal in ii, iii. (iv) Histochemical localization of *FT* promoter-GUS in a cleared inflorescence 22-day-old-plant grown under LDs. The plant was harvested at ZT16. Blue arrowheads highlight GUS accumulation within the vascular tissue in the peduncle; orange arrowheads mark GUS staining in the vascular tissue of the pedicels. Scale bars in i, iii, and iv = 50 μm; in ii = 100 μm.

Development • Supplementary information

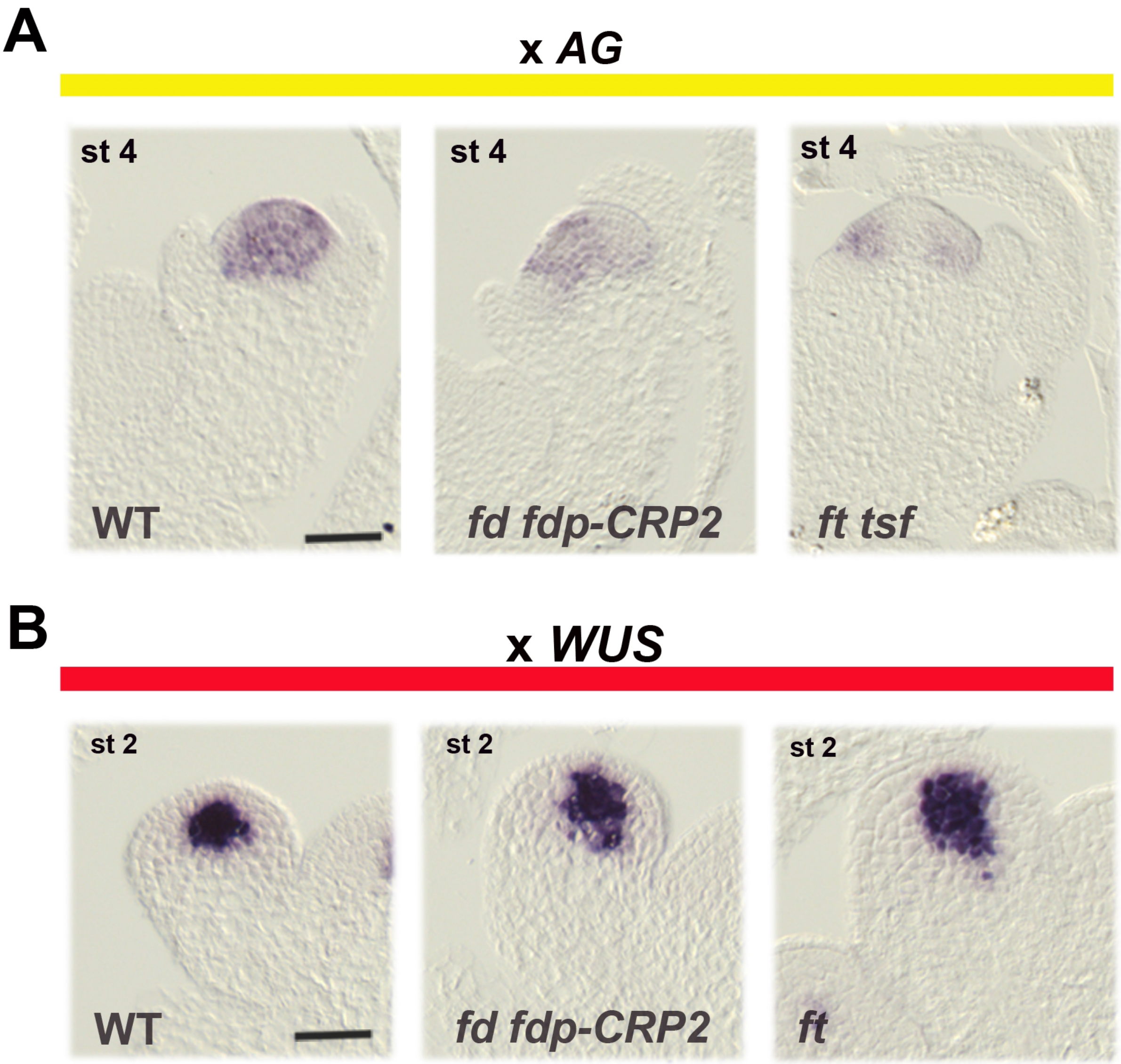

Fig. S6. Related to Fig. 7. The mRNA of *AG* is downregulated in the floral buds of *fd fdp* and *ft tsf* double mutants, whereas *WUS* mRNA is more highly expressed. *In situ* hybridization of *AG* (A) and *WUS* (B) mRNAs in floral buds of the depicted genotypes grown for 22 long days (LDs) for WT, 24 LDs for *fd-3 fdp-CRP2*, 29 LDs for *ft-10* or 34 LDs for *ft-10 tsf-1*. Images show flower buds at stage 4 (A) and stages 2 (B). Scale bars in all panels correspond to 50 µm

Development • Supplementary information

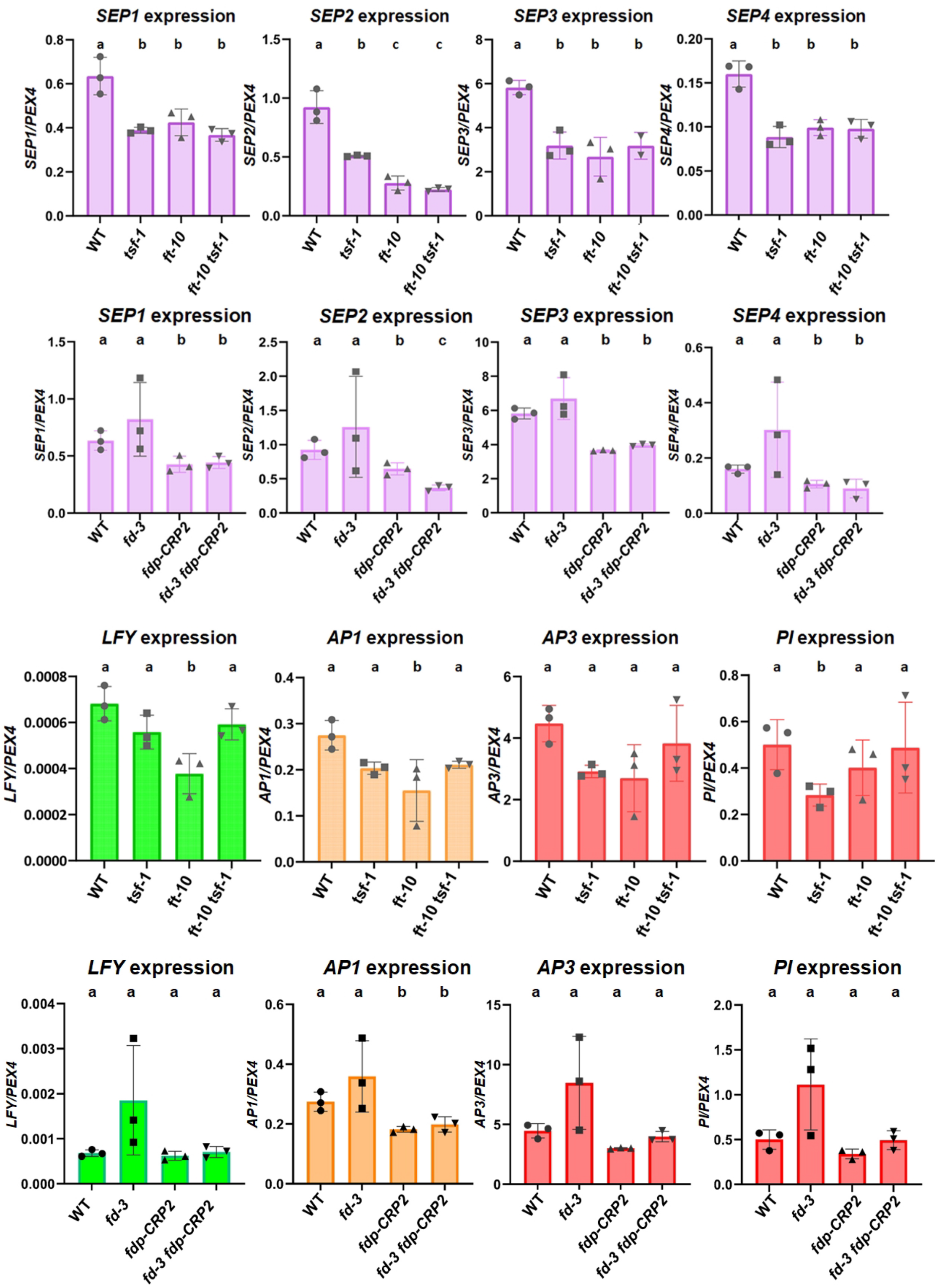

Fig. S7. Related to Figure 7. mRNA levels of floral meristem identity and floral identity genes in WT, *ft, tsf, fd*, *fdp*, *ft tsf* and *fd fdp*. RT-qPCR analysis of *SEP1–SEP4*, *LEAFY* (*LFY*), *APETALA 1* (*AP1*), *APETALA 3* (*AP3*) and *PISTILLATA* (*PI*) mRNA abundance in inflorescences of wild type (WT), *tsf-1, ft-10* and *ft-10 tsf-1* and WT, *fd-3, fdp-CRP2* and *fd-3 fdp-CRP2*. Inflorescences were harvested 6 days after the first flower opened in all genotypes. Data are means ± SD of three biological replicates. Common letters among genotypes indicate no significant differences in mRNA levels (one-way ANOVA with Tukey's test; $P \leq 0.05$).

Development • Supplementary information

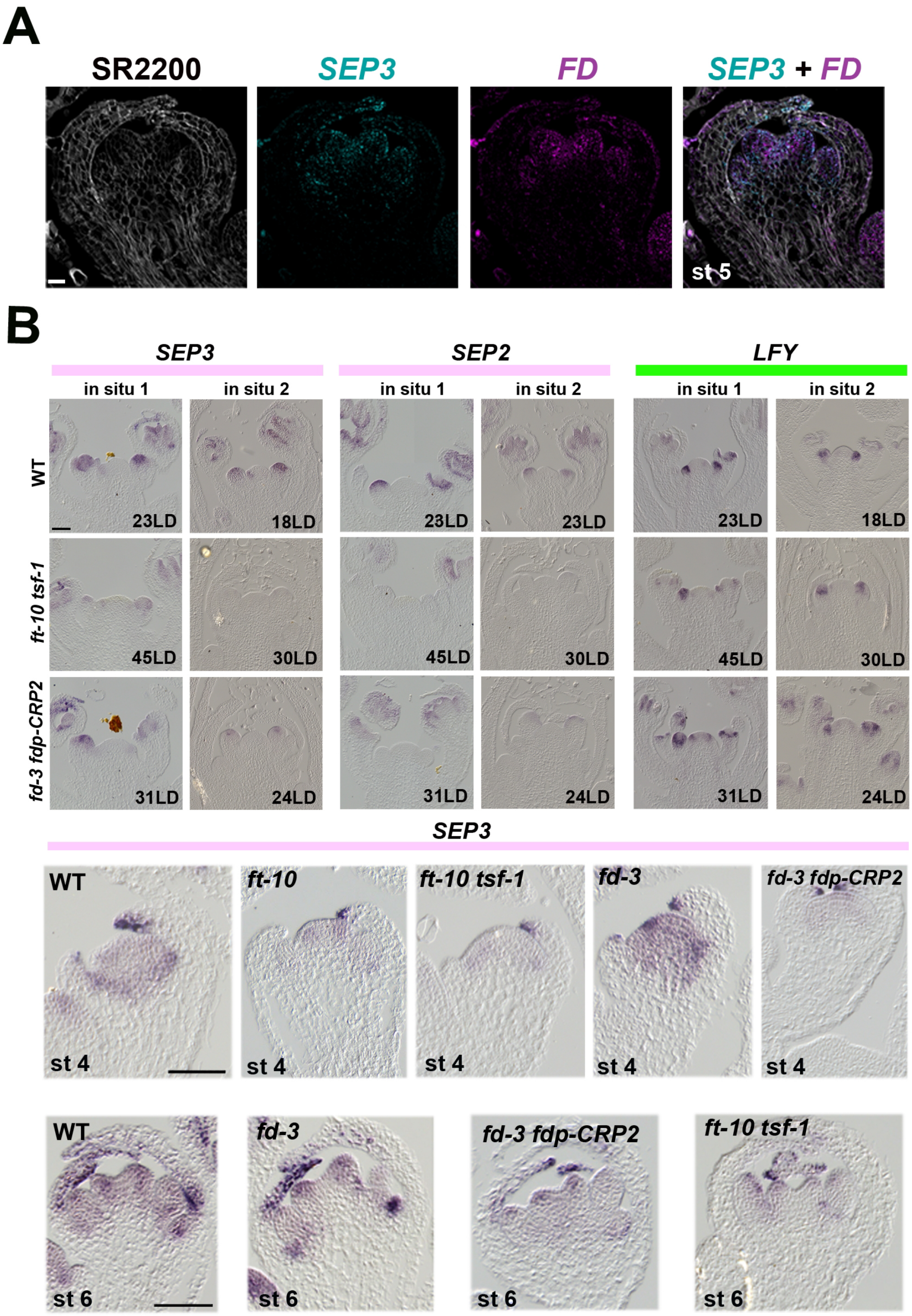
A
SR2200
SEP3
FD
SEP3 + FD
st 5
B
SEP3
SEP2
LFY
in situ 1
in situ 2
in situ 1
in situ 2
in situ 1
in situ 2
WT
23LD
18LD
23LD
23LD
23LD
18LD
ft-10 tsf-1
45LD
30LD
45LD
30LD
45LD
30LD
fd-3 fdp-CRP2
31LD
24LD
31LD
24LD
31LD
24LD
SEP3
WT
ft-10
ft-10 tsf-1
fd-3
fd-3 fdp-CRP2
st 4
st 4
st 4
st 4
st 4
WT
fd-3
fd-3 fdp-CRP2
ft-10 tsf-1
st 6
st 6
st 6
st 6

Fig. S8. Related to Fig. 7. Co-localization of *FD* and *SEP3* mRNAs by RNAscope and expression pattern analysis of *SEP2*, *SEP3*, and *LFY* by *in situ* hybridization in the inflorescence of WT *fd, fd fdp, ft*, and *ft tsf* mutants.
(A) RNAscope *in situ* hybridizations of *SEP3* and *FD* mRNAs in inflorescences of Col-0. SCRI Renaissance 2200 stain cell walls in white. *SEP3* and *FD* mRNAs appear in the depicted colours. Flower bud shown is at stage 5, as indicated. Plants were grown under 18LD conditions. (B) Upper panels: *in situ* hybridizations of *SEPALLATA 2* (*SEP2*), (*SEP3*) and *LEAFY* (*LFY*) mRNAs in inflorescences of the depicted genotype s from plants grown in long days (LDs) for the times indicated. Two independent *in situ* hybridizations are shown (*in situ* 1 & *in situ* 2). Lower panels: floral buds at stage 4 and 6 of the genotypes indicated. Plants were grown for 24 LDs for WT, 33 LDs for *fd-3 fdp-CRP2*, 35 LDs for *ft-10*, 28 or 32 LDs for *fd-3*, 45 LDs for *ft-10 tsf-1*. Scale bar A= 20 µm; B = 50 µm and applies to all panels.

Table S1. Quantification of floral organ number in long days for genotypes using Col-0 background

Available for download at
https://journals.biologists.com/dev/article-lookup/doi/10.1242/dev.204241#supplementary-data

Table S2. Pedicel width

Available for download at
https://journals.biologists.com/dev/article-lookup/doi/10.1242/dev.204241#supplementary-data

Table S3. Flowering time under short days

Available for download at
https://journals.biologists.com/dev/article-lookup/doi/10.1242/dev.204241#supplementary-data

Table S4. Quantification of floral organ number in short days for genotypes using Col-0 background

Available for download at
https://journals.biologists.com/dev/article-lookup/doi/10.1242/dev.204241#supplementary-data

Development • Supplementary information

Table S5. Quantification of floral organ number in long days for genotypes us ing L*er* background

Available for download at
https://journals.biologists.com/dev/article-lookup/doi/10.1242/dev.204241#supplementary-data

Table S6. 74 genes bound by FD and MADS-box transcription factors.

Available for download at
https://journals.biologists.com/dev/article-lookup/doi/10.1242/dev.204241#supplementary-data

Table S7. List of primers used in this study

Available for download at
https://journals.biologists.com/dev/article-lookup/doi/10.1242/dev.204241#supplementary-data
