# Supplementary material for: FD and FDP bZIP transcription factors and FT florigen regulate floral development and control homeotic gene expression in Arabidopsis floral meristems
Source: Development. 2025 May 22;152(10):dev204241. doi: 10.1242/dev.204241 (PMC12148031; doi:10.1242/dev.204241)
Supplement: Supplementary file 1 [file develop-152-204241_review_history.pdf]

FD and FDP bZIP transcription factors and FT florigen regulate floral development and control homeotic gene expression in *Arabidopsis* floral meristems

Maida Romera-Branchat, Chloé Pocard, Coral Vincent, Martina Cerise, Vıt́or da Silveira Falavigna, Alice Pajoro, Na Ding, He Gao, Rainer Franzen and George Coupland
DOI: 10.1242/dev.204241

Editor: Dominique Bergmann

Review timeline
Original submission: 22 July 2024
Editorial decision: 23 August 2024
First revision received: 13 March 2025
Accepted: 12 April 2025

Original submission

First decision letter

MS ID#: dev.204241

MS TITLE: FD/FDP bZIP transcription factors and FT florigen regulate floral patterning and control homeotic gene expression in Arabidopsis floral meristems

AUTHORS: Maida Romera-Branchat, George Coupland, Chloé Pocard, Coral Vincent, Martina Cerise, Vitor Falavigna, Alice Pajoro, He Gao and Rainer Franzen

Dear Dr Coupland,

I have now received all the referees' reports on the above manuscript, and have reached a decision. The referees' comments are appended below, or you can access them online: please go to:

As you will see, the referees express considerable interest in your work, but have some significant criticisms and recommend a substantial revision of your manuscript before we can consider publication. If you are able to revise the manuscript along the lines suggested, which may involve further experiments, I will be happy receive a revised version of the manuscript. Your revised paper will be re-reviewed by one or more of the original referees, and acceptance of your manuscript will depend on your addressing satisfactorily the reviewers' major concerns. Please also note that Development will normally permit only one round of major revision.

As you will see from the detailed reviews below, there was some divergence in the overall assessment of your work, with reviewer 1 having several points they felt needed additional experiments and/or modifications to the text. I strongly urge you to send us a point-by-point response indicating your plans for addressing the referees' comments, and we will look over this and provide further guidance.

Reviewer 1

Advance summary and potential significance to field

© 2025. Published by The Company of Biologists under the terms of the Creative Commons Attribution License (https://creativecommons.org/licenses/by/4.0/).

The work presented by Romera-Branchat et al. focuses on describing the role of FD/FDP and FT/TSF during floral development, particularly their effect on the homeotic genes expression and the consequent defects in the organ patterning within the flowers. The authors described the expression patterns of FD, FDP and FT, but not TFS at different stages of floral development and carried out a deep morphological characterization of different mutant combinations, analyzing several parameters. Finally, they showed that SEP homeotic genes are downregulated in the mutant backgrounds of interest, suggesting a role for FD/FDP and FT/TSF in the regulation of these genes. The paper presents a substantial amount of well-organized data, the morphological characterization of the flowers throughout the early stages of development (0-6) however, the work lacks novelty and not all the conclusions described in the discussion section are well supported by the experimental procedures. In particular, the distinct and overlapping roles of the two sets of genes FD/FDP and FT/TSF are not always illustrated convincingly. For instance, the pattern of expression of these four proteins does not overlap with the ones of the SEP genes which are described as their direct targets, nor with AG. Additionally, while the authors have documented various morphological defects and misregulation of target genes, they fail to coherently link these findings to the broader model of flower development. Overall, the study is largely qualitative and does not contribute significantly to advancing the current understanding of floral gene regulation.

Comments for the author

* The authors deeply characterized the floral phenotypes of fd and fdp single and double mutants, as well as the ones of ft and tsf. However, the similarity between the phenotypes observed in fd, fdp and ft, tsf is not obvious, especially when looking at the sepal/petal number which appears to be much lower in the ft tsf. If possible, the authors should discuss in more detail the comparison between the phenotypes of these two groups of genes, underlining the similarities which led to the hypothesis that these genes share a similar function during flower development.
* In Figure 1A, the authors define the expression of FD mRNA as absent at stage 1, but in this section, I can detect the signal in Figure 1Ai. In the description of VENUS:FD expression profile, the authors wrote "was absent at stage 1 but reappeared at stage 2 in the epidermis", I would change it to abaxial region of the floral primordia. Expression in sepals should be described as confined at the base of sepals at stage 4. In stage 6 the expression in sepals is not convincing and petals primordia are not present in this image 1Cii. Co-localization of these bZIP TFs was tested using plants containing both FDP::VENUS:FDP and FD::mCHERRY:FD. The overlapping of these factors could suggest a cooperative role in these developmental stages, not necessarily that they are redundant. Moreover, overlapping in the lower part of the floral meristem at early stage 3 shown in Fig 1Di, doesn't support their role as direct regulators of floral homeotic genes and in particular AG.
* The authors should explain why they used the two mutant alleles fdp-2 and fdp-CRP2.
* The authors suggest that in the ft-10 tsf-1 and fd-3 fdp-CRP2 mutants the observed enlargement of the pedicel width is a direct consequence of the enlargement of the floral meristem. However, the measure of the pedicel was performed at anthesis while the floral meristem was measured at a much earlier stage (3-4). Is the increase in the floral meristem maintained during later stages of development or are the floral organs enlarged in these mutants? Furthermore, pedicels of ft-10 tsf-1 do not differ in size from the wild type, even if at stage 3 their floral meristems appear to be enlarged, suggesting that floral meristem size in this case does not influence pedicel width. It would be also interesting to analyse the number and the dimension of the cells in the floral domes at stage 3 to better define the role these players have in controlling the dimension of the reproductive meristems.
* The authors linked the increased floral meristem size to the prolonged and stronger expression of WUS in fd fdp-CRP2 and ft mutants. Since the ft tsf mutant is not affected in the meristem size, to further prove the direct correlation between floral meristem size and WUS expression, it would be nice to perform the WUS in situ hybridization on this mutant as well.
* The authors hypothesize that FT/FD directly regulates the expression of SEP genes. Even if the decrease in SEPs mRNA is clearly shown by both in situ and real-time PCR, the expression patterns of the SEPs and especially FT do not seem to overlap in the stages shown in Figure 7 and Figure 3 suggesting that an indirect regulation is more likely.
* A sort of complementation test could strengthen the finding of the research, for example, the expression of SEP3 in the fd fdp-CRP2 and ft tsf under the control of a reproductive meristems promoter not bound by these players.

Minor suggestions.

© 2025. Published by The Company of Biologists under the terms of the Creative Commons Attribution License (https://creativecommons.org/licenses/by/4.0/).

- In the introduction, the function and development of the axillary meristems is not very clear. The phrase "and axillary meristems transition to floral primordia that contain floral meristems" is not precise, I would use identity instead of that transition, moreover, the floral primordia is itself a floral meristem.
- Full names for the genes should be inserted at the beginning of the introduction.
- To make the manuscript more easily understandable, authors should explain the difference between the FT::FT:GFP and SUC2::FT:GFP lines
- The genomic binding sites of FD and FDP, as well as several MADS-domain TFs contributing to floral development, have been identified through ChIP-seq (Kaufmann et al., 2009; Kaufmann et al., 2010; Wuest et al., 2012). Ref for FD and FDP genomic binding sites is missing.

Reviewer 2

Advance summary and potential significance to field

The manuscript by Romera-Branchat et al from the lab of George Coupland reports on the FD/FDP bZIP transcription factors and FT/TSF florigens regulating floral patterning and control homeotic gene expression in Arabidopsis floral meristems. Using genetics, imaging, in situ hybridizations, and comparisons of ChIP-seq data-sets, evidently the results demonstrate that FD/FDP and FT/TSF are involved in flower development, besides their function in induction of flowering.
All results are clearly described, experiments are of the highest quality, and figures beautiful. This work is a great contribution to the current knowledge on flower development. I loved reading the manuscript and there is nothing to comment on this work.

First revision

Author response to reviewers' comments

Response to reviewers
We would like to thank the editor and reviewers for considering our manuscript, and for their valuable comments on the text and data. We respond to each of their comments below. Our responses are in bold and the reviewers' and editor's comments appear verbatim. We have provided line numbers in the new document for the relevant changes, because there were too many alterations to the text to mark them using track changes.
Editor
As you will see, the referees express considerable interest in your work, but have some significant criticisms and recommend a substantial revision of your manuscript before we can consider publication. If you are able to revise the manuscript along the lines suggested, which may involve further experiments, I will be happy receive a revised version of the manuscript. Your revised paper will be re-reviewed by one or more of the original referees, and acceptance of your manuscript will depend on your addressing satisfactorily the reviewers' major concerns. Please also note that Development will normally permit only one round of major revision.
As you will see from the detailed reviews below, there was some divergence in the overall assessment of your work, with reviewer 1 having several points they felt needed additional experiments and/or modifications to the text. I strongly urge you to send us a point-by-point response indicating your plans for addressing the referees' comments, and we will look over this and provide further guidance.

Please attend to all of the reviewers' comments and ensure that you clearly highlight all changes made in the revised manuscript. Please avoid using 'Tracked changes' in Word files as these are lost in PDF conversion. I should be grateful if you would also provide a point-by-point response detailing how you have dealt with the points raised by the reviewers in the 'Response to Reviewers' box. If you do not agree with any of their criticisms or suggestions please explain clearly why this is so.

© 2025. Published by The Company of Biologists under the terms of the Creative Commons Attribution License (https://creativecommons.org/licenses/by/4.0/).

We appreciate the editor's invitation to submit a revised version that incorporates new data and modifications to the text in response to the comments of Reviewer 1. We have provided a point-by-point response to their comments below.

Reviewer 1:
SUMMARY OF THE ADVANCE MADE IN THIS PAPER AND ITS POTENTIAL SIGNIFICANCE TO THE FIELD

The work presented by Romera-Branchat et al. focuses on describing the role of FD/FDP and FT/TSF during floral development, particularly their effect on the homeotic genes expression and the consequent defects in the organ patterning within the flowers. The authors described the expression patterns of FD, FDP and FT, but not TFS at different stages of floral development and carried out a deep morphological characterization of different mutant combinations, analyzing several parameters. Finally, they showed that SEP homeotic genes are downregulated in the mutant backgrounds of interest, suggesting a role for FD/FDP and FT/TSF in the regulation of these genes. The paper presents a substantial amount of well-organized data, the morphological characterization of the flowers throughout the early stages of development (0-6) however, the work lacks novelty and not all the conclusions described in the discussion section are well supported by the experimental procedures. In particular, the distinct and overlapping roles of the two sets of genes FD/FDP and FT/TSF are not always illustrated convincingly. For instance, the pattern of expression of these four proteins does not overlap with the ones of the SEP genes which are described as their direct targets, nor with AG. Additionally, while the authors have documented various morphological defects and misregulation of target genes, they fail to coherently link these findings to the broader model of flower development. Overall, the study is largely qualitative and does not contribute significantly to advancing the current understanding of floral gene regulation.

SUGGESTIONS TO AUTHORS

* The authors deeply characterized the floral phenotypes of fd and fdp single and double mutants, as well as the ones of ft and tsf. However, the similarity between the phenotypes observed in fd, fdp and ft, tsf is not obvious, especially when looking at the sepal/petal number which appears to be much lower in the ft tsf. If possible, the authors should discuss in more detail the comparison between the phenotypes of these two groups of genes, underlining the similarities which led to the hypothesis that these genes share a similar function during flower development.
We appreciate these suggestions to improve the text. We have provided new data and edited the text in response to the two major points raised by the reviewer. (1) The reviewer points out that there are differences as well as similarities in the phenotypes of the single and double mutants. We agree that in the original submission we oversimplified the roles of these two pairs of genes and argued too strongly in the text that they have the same functions in all contexts. Indeed, as the reviewer points out the floral phenotype of *ft tsf* mutants is stronger than that of *fd fdp* and that in particular *ft tsf* has fewer petals. We make this point now explicitly in the Results on lines 187-194 of the new version, and mention specifically petal number on line 191-194. In the Results, the floral phenotypes of *fd fdp* and *ft tsf* mutants are presented in different figures (2 and 3, respectively), so in the revised version, we have also included one histogram showing the number of sepals and petals in the flowers of the different genotypes, which allows the effects of the mutations to be more easily directly compared (Figure S3). In the Discussion on lines 449-452, we mention that a stronger phenotype of *ft tsf* than *fd fdp* is also observed in flowering time, and that this is usually explained by further layers of redundancy in bZIP TFs causing a weaker *fd fdp* phenotype, which might also explain the stronger effect of *ft tsf* on floral organ number. However, in terms of floral meristem size, the effects of *ft tsf* and *fd fdp* are almost identical (new Figure 5). Also, the effects of *ft tsf* and *fd fdp* on *SEP*, *AG* and *WUS* gene expression in the floral meristem are very similar (new Figure 7), although again *ft tsf* has a slightly stronger effect in floral primordia when measured by RT-qPCR, as mentioned on lines 330-331. The similarities in these floral meristem phenotypes lead us to the conclusion that FT TSF and FD FDP share common roles in the regulation of floral meristem size. In the Discussion, we have stressed that the roles in floral meristem size regulation are similar, but in other aspects of floral development their involvement might differ and FT TSF has a generally stronger effect on floral organ development (e.g. Lines 449-451). (2) The second point is to coherently link the roles of FT TSF and FD FDP to current models of floral development. We have stressed the similar contributions of FD FDP and FT TSF to the regulation of floral meristem size, and the

© 2025. Published by The Company of Biologists under the terms of the Creative Commons Attribution License (https://creativecommons.org/licenses/by/4.0/).

demonstration that they are required for the activation of *SEP2*, *SEP3* and *AG*, and the repression of *WUS* expression in the floral meristem. These points incorporate these genes into current models of the regulation of floral meristem size. We are nevertheless clear that we cannot explain all of the floral organ phenotypes at this stage (Discussion line 472-474). We do not agree that this work does not advance "the current understanding of floral gene regulation" as it places FD FDP and their interacting proteins FT TSF as direct regulators of *SEP* genes in the floral meristem, and links these to the activation of *AG* and repression of *WUS*. We believe that these will be influential observations, defining regulatory roles for these flowering-time regulators in the floral meristem.

* In Figure 1A, the authors define the expression of FD mRNA as absent at stage 1, but in this section, I can detect the signal in Figure 1Ai. In the description of VENUS:FD expression profile, the authors wrote "was absent at stage 1 but reappeared at stage 2 in the epidermis", I would change it to abaxial region of the floral primordia. Expression in sepals should be described as confined at the base of sepals at stage 4. In stage 6 the expression in sepals is not convincing and petals primordia are not present in this image 1Cii. Co-localization of these bZIP TFs was tested using plants containing both FDP::VENUS:FDP and FD::mCHERRY:FD. The overlapping of these factors could suggest a cooperative role in these developmental stages, not necessarily that they are redundant. Moreover, overlapping in the lower part of the floral meristem at early stage 3 shown in Fig 1Di, doesn't support their role as direct regulators of floral homeotic genes and in particular AG.
We have carefully considered the points raised by the reviewer on the gene expression data and how we have described it. In the revised version, we have added new data and have edited our interpretations accordingly.

- **We have repeated the *FD in situ* in Figure 1A and replaced the image with one that more clearly shows *FD* expression in stage 1 flowers. This new image shows that *FD* mRNA is detected at the base of stage 1 primordia, and we have added this interpretation on line 117-118.**
- **We have edited the description of the expression patterns in stage 2 flowers and stage 4 sepals as suggested by the reviewer (on lines 126-129).**
- **We have included new confocal images of older flowers (Figure 1C iii, vi). These clearly show VENUS-FD throughout the petal primordium and VENUS-FDP on the adaxial side of the petal primordium.**
- **We have deleted the sentence at the end of the Results section stressing redundancy, because as the reviewer points out the expression pattern alone is not sufficient to make this point.**
- **In terms of the floral meristem, it is true that the overlap between the FD and FDP expression patterns is limited to the lower part of the floral meristem. The gene expression changes detected in the floral meristem in *fd fdp* may be mainly caused by *fd*. We have now shown that *SEP3* and *FD* mRNAs overlap fully in the stage 3 floral meristem (Figure 7C). Also, we have now tested *WUS* mRNA expression in the *fd* single mutant and show that even in this genotype *WUS* expression is increased at stage 7 (Figure 7Bv) compared to Col-0, suggesting that the *fd* mutation is already sufficient to cause the reduced *SEP* and *AG* expression resulting in increased *WUS* described in *fd fdp*. Nevertheless, FDP likely contributes to the reduced *SEP* expression in the lower floral meristem where FDP and FD overlap, and in other organs where FDP is expressed in older flowers (Figure 1C) because the RT-qPCR data clearly showed that *SEP2* and *SEP3* are more strongly reduced in expression in *fd fdp* than in *fd* mutants (Figure S7), and *in situ* hybridizations showed that *AG* mRNA was more strongly reduced in floral organs of *fd fdp* than *fd* (Figure 7Aiv-vi).**

* The authors should explain why they used the two mutant alleles fdp-2 and fdp-CRP2.

Previously, we showed that *fdp-CRP2* is a null allele, but that *fdp-2* has a stronger flowering phenotype, probably because it has a dominant negative effect on other bZIPs due to a missense mutation in the DNA binding region (Romera-Branchat et al, 2020). Therefore, we usually include both alleles in our analyses. We have now mentioned the difference between these alleles on line 145-147.
* The authors suggest that in the ft-10 tsf-1 and fd-3 fdp-CRP2 mutants the observed enlargement of the pedicel width is a direct consequence of the enlargement of the floral meristem. However, the measure of the pedicel was performed at anthesis while the floral meristem was measured at a

© 2025. Published by The Company of Biologists under the terms of the Creative Commons Attribution License (https://creativecommons.org/licenses/by/4.0/).

much earlier stage (3-4). Is the increase in the floral meristem maintained during later stages of development or are the floral organs enlarged in these mutants? Furthermore, pedicels of ft-10 tsf-1 do not differ in size from the wild type, even if at stage 3 their floral meristems appear to be enlarged, suggesting that floral meristem size in this case does not influence pedicel width. It would be also interesting to analyse the number and the dimension of the cells in the floral domes at stage 3 to better define the role these players have in controlling the dimension of the reproductive meristems.
The reviewer is correct that we measured meristem size at an earlier stage than pedicel width and that the *ft tsf* mutant has an increased meristem size but no effect on pedicels. It is also clear that these genes are expressed directly in pedicels, as mentioned on line 130, 134 and line 276. Therefore, we have removed the implication that the pedicel width phenotype is a consequence of the floral meristem phenotype. We mention the pedicel phenotype as part of the description of these mutants (*fd-3 fdp-CRP2*, line 164-168; *ft-10 tsf-1* line 208-211), but separate it from the section describing floral meristems (from line 247). We have not been able in the time available for these revisions to segment the floral meristems of these mutants at stage 3 to compare cellular dimensions. However, the tissue level measurements of width and height are shown in detail (Figure 5).
* The authors linked the increased floral meristem size to the prolonged and stronger expression of WUS in fd fdp-CRP2 and ft mutants. Since the ft tsf mutant is not affected in the meristem size, to further prove the direct correlation between floral meristem size and WUS expression, it would be nice to perform the WUS in situ hybridization on this mutant as well.
The reviewer is right that we previously showed *WUS* expression in *ft* but not *ft tsf*. We have now done that and show in Figure 7B that *WUS* expression is also increased at stage 6 and stage 8 in *ft tsf*. This correlates with the larger floral meristem of *ft tsf* (Figure 5). The reviewer might have transposed the effect of *ft tsf* on pedicel width and floral meristem size - it does have a larger floral meristem but does not have a wider pedicel.
* The authors hypothesize that FT/FD directly regulates the expression of SEP genes. Even if the decrease in SEPs mRNA is clearly shown by both in situ and real-time PCR, the expression patterns of the SEPs and especially FT do not seem to overlap in the stages shown in Figure 7 and Figure 3 suggesting that an indirect regulation is more likely.
The pattern of *SEP2* and *SEP3* mRNAs overlap well with that of FD in the floral meristem. We have now shown that for *SEP3* and *FD* mRNAs by RNAscope in the same tissue sections (Figure 7C). The ChIPseq of FD is also consistent with direct regulation of the SEP genes (Figure 8, and previous publications). However, we have not been able to obtain an *in situ* of *FT* mRNA in the stage 3-4 flower. We did include *FT* probes in the same RNAscope experiment that we did for *SEP3/FD* and obtained a broad, weak signal, which we are not sufficiently confident of to publish. However, based on this, we suspect that *FT* mRNA could be widely and weakly expressed in stage 3-4 flowers. Nevertheless, in *FT:FT-GFP* plants we detected FT-GFP protein in the floral meristem of stage 3-4 flowers (Figure 6) and this construct complemented the floral phenotype. *FT* has been shown to be expressed in the phloem companion cells and to control floral transition there, however we found that when expressed from the *SUC2* promoter that is specific to companion cells, the flowering-time phenotype was complemented, but the flower phenotype was not and no FT-GFP was detected in floral meristems (Figure 6). We conclude from this experiment that the *FT* promoter is likely to be active in stage 3-4 flowers and this is responsible for the expression of FT-GFP in floral meristems, but that the mRNA level is so low we cannot detect it by *in situ* hybridization or RNAscope. We describe these data in the Results on line 269-290 and have expanded this in the new Discussion on line 421-436.
* A sort of complementation test could strengthen the finding of the research, for example, the expression of SEP3 in the fd fdp-CRP2 and ft tsf under the control of a reproductive meristems promoter not bound by these players.
This is a logical experiment however we could not make and score new transgenic plants in the time available to us. Also, we found that *SEP1*, *2*, *3*, and *4* were reduced in the *ft tsf* double mutant so it is possible that heterologous expression of one of these genes would not be sufficient. However, we have mentioned this experiment as a future test of our model in the Discussion (lines 501-503).
Minor suggestions.

- In the introduction, the function and development of the axillary meristems is not very clear. The phrase "and axillary meristems transition to floral primordia that contain floral meristems" is not

© 2025. Published by The Company of Biologists under the terms of the Creative Commons Attribution License (https://creativecommons.org/licenses/by/4.0/).

precise, I would use identity instead of that transition, moreover, the floral primordia is itself a floral meristem.

We have edited this point as suggested by the reviewer (line 49-50).
- Full names for the genes should be inserted at the beginning of the introduction.

We have included the full name of FDP (line 58-59), FD does not have a longer name because it was named FD from its original description, and all of the other genes seem to be already mentioned with their full names in the Introduction (e.g. line 62, lines 69-71).
- To make the manuscript more easily understandable, authors should explain the difference between the FT::FT:GFP and SUC2::FT:GFP lines
We mentioned the difference between these lines above, and have tried to make it clearer in the text in the Results (line 269-290) and in the Discussion (line 421-430).
- The genomic binding sites of FD and FDP, as well as several MADS-domain TFs contributing to floral development, have been identified through ChIP-seq (Kaufmann et al., 2009; Kaufmann et al., 2010; Wuest et al., 2012). Ref for FD and FDP genomic binding sites is missing.
We have restructured and shortened this section, so the precise sentence mentioned by the reviewer no longer appears. However, we now have a sentence at the beginning of this section that cites these papers: "The genomic binding sites of FD and FDP were identified by ChIPseq (Collani et al., 2019; Romera-Branchat et al., 2020; Zhu et al., 2020)." (Line 370-371). Also, the Methods section describes the data set used for the comparisons (Lines 679-680).

Reviewer 2: The manuscript by Romera-Branchat et al from the lab of George Coupland reports on the FD/FDP bZIP transcription factors and FT/TSF florigens regulating floral patterning and control homeotic gene expression in Arabidopsis floral meristems. Using genetics, imaging, in situ hybridizations, and comparisons of ChIP-seq data-sets, evidently the results demonstrate that FD/FDP and FT/TSF are involved in flower development, besides their function in induction of flowering.
All results are clearly described, experiments are of the highest quality, and figures beautiful. This work is a great contribution to the current knowledge on flower development. I loved reading the manuscript and there is nothing to comment on this work.
We thank the reviewer for their interest in our work and for their appreciation of the data.

Second decision letter

MS ID#: dev.204241

MS TITLE: FD/FDP bZIP transcription factors and FT florigen regulate floral patterning and control homeotic gene expression in Arabidopsis floral meristems

AUTHORS: Maida Romera-Branchat, George Coupland, Chloé Pocard, Coral Vincent, Martina Cerise, Vitor Falavigna, Alice Pajoro, Na Ding, He Gao and Rainer Franzen

Dear Dr Coupland,

I am happy to tell you that your manuscript has been accepted for publication in Development, pending our standard publication integrity checks.

Reviewer 1

Advance summary and potential significance to field

The work presented by Romera-Branchat et al. focuses on the role of FD/FDP and FT/TSF during floral development, particularly their effect on the expression of homeotic genes and the resulting defects in floral organ patterning. The authors describe the expression patterns of FD, FDP, and FT,

© 2025. Published by The Company of Biologists under the terms of the Creative Commons Attribution License (https://creativecommons.org/licenses/by/4.0/).

but not TSF at different stages of floral development and perform a detailed morphological characterization of various mutant combinations, analyzing multiple parameters. Finally, they show that SEP homeotic genes are downregulated in the mutant backgrounds of interest, suggesting a role for FD/FDP and FT/TSF in the regulation of these genes.

The paper presents a substantial amount of well-organized data, including a morphological characterization of flowers during the early stages of development (stages 0-6), along with new and convincing data and images that support their conclusions.

In particular, the distinct and overlapping roles of the two gene sets FD/FDP and FT/TSF are now convincingly illustrated. The authors now better correlate the effects on pedicel and floral meristem size with the different mutants. As proposed, they also clarify the correlation between WUS expression and meristem size in the ft tsf mutant background.

Comments for the author

I truly appreciate the effort made in addressing almost all of my concerns. I understand that some experiments could not be performed due to time constraints, and others for purely technical reasons, nevertheless I belive that the revised version of the manuscript better presents the authors' hypotheses and more clearly articulates their conclusions.

<u>Reviewer 2</u>

Comments for the author

The authors made good improvements to the manuscript. I do not have further comments.

© 2025. Published by The Company of Biologists under the terms of the Creative Commons Attribution License (https://creativecommons.org/licenses/by/4.0/).
